# Immune cells: potential carriers or agents for drug delivery to the central nervous system

**DOI:** 10.1186/s40779-024-00521-y

**Published:** 2024-03-29

**Authors:** Shan-Shan Zhang, Ruo-Qi Li, Zhong Chen, Xiao-Ying Wang, Aaron S. Dumont, Xiang Fan

**Affiliations:** 1 School of Basic Medical Sciences, Zhejiang Chinese Medical University, No. 548 Binwen Road, Binjiang District, Hangzhou, 310053 China; 2 Key Laboratory of Neuropharmacology and Translational Medicine of Zhejiang Province, Zhejiang Chinese Medical University, Hangzhou, 310053 Zhejiang China; 3 Clinical Neuroscience Research Center, Department of Neurosurgery and Neurology, Tulane University School of Medicine, New Orleans, LA 70122 USA

**Keywords:** Drug delivery systems, Immune cells, Blood–brain barrier, Central nervous system

## Abstract

Drug delivery systems (DDS) have recently emerged as a promising approach for the unique advantages of drug protection and targeted delivery. However, the access of nanoparticles/drugs to the central nervous system (CNS) remains a challenge mainly due to the obstruction from brain barriers. Immune cells infiltrating the CNS in the pathological state have inspired the development of strategies for CNS foundation drug delivery. Herein, we outline the three major brain barriers in the CNS and the mechanisms by which immune cells migrate across the blood–brain barrier. We subsequently review biomimetic strategies utilizing immune cell-based nanoparticles for the delivery of nanoparticles/drugs to the CNS, as well as recent progress in rationally engineering immune cell-based DDS for CNS diseases. Finally, we discuss the challenges and opportunities of immune cell-based DDS in CNS diseases to promote their clinical development.

## Background

According to global statistics, central nervous system (CNS) diseases, such as gliomas, stroke, Alzheimer’s disease (AD), Parkinson’s disease (PD), multiple sclerosis (MS), epilepsy, and several others diseases, are the leading causes of disability worldwide, accounting for approximately 12% of total deaths. Among these conditions, AD and PD stand out as the most prevalent neurodegenerative diseases [1]; meanwhile, stroke ranks second in terms of mortality rates [2]. Therefore, there is an urgent demand for enhanced management and treatment strategies for CNS diseases. However, due to the presence of CNS barriers, nearly 98% of small-molecule drugs and all large-molecule drugs are routinely excluded from reaching the brain [3]. This predicament has forced researchers to explore more effective treatments. Nevertheless, the lack of success in most preclinical and clinical studies conducted so far has highlighted great challenges in the clinical application of drugs targeting CNS diseases, including inadequate drug selectivity, limited permeability across the low blood–brain barrier (BBB), and/or rapid elimination.

**Fig. 1:**
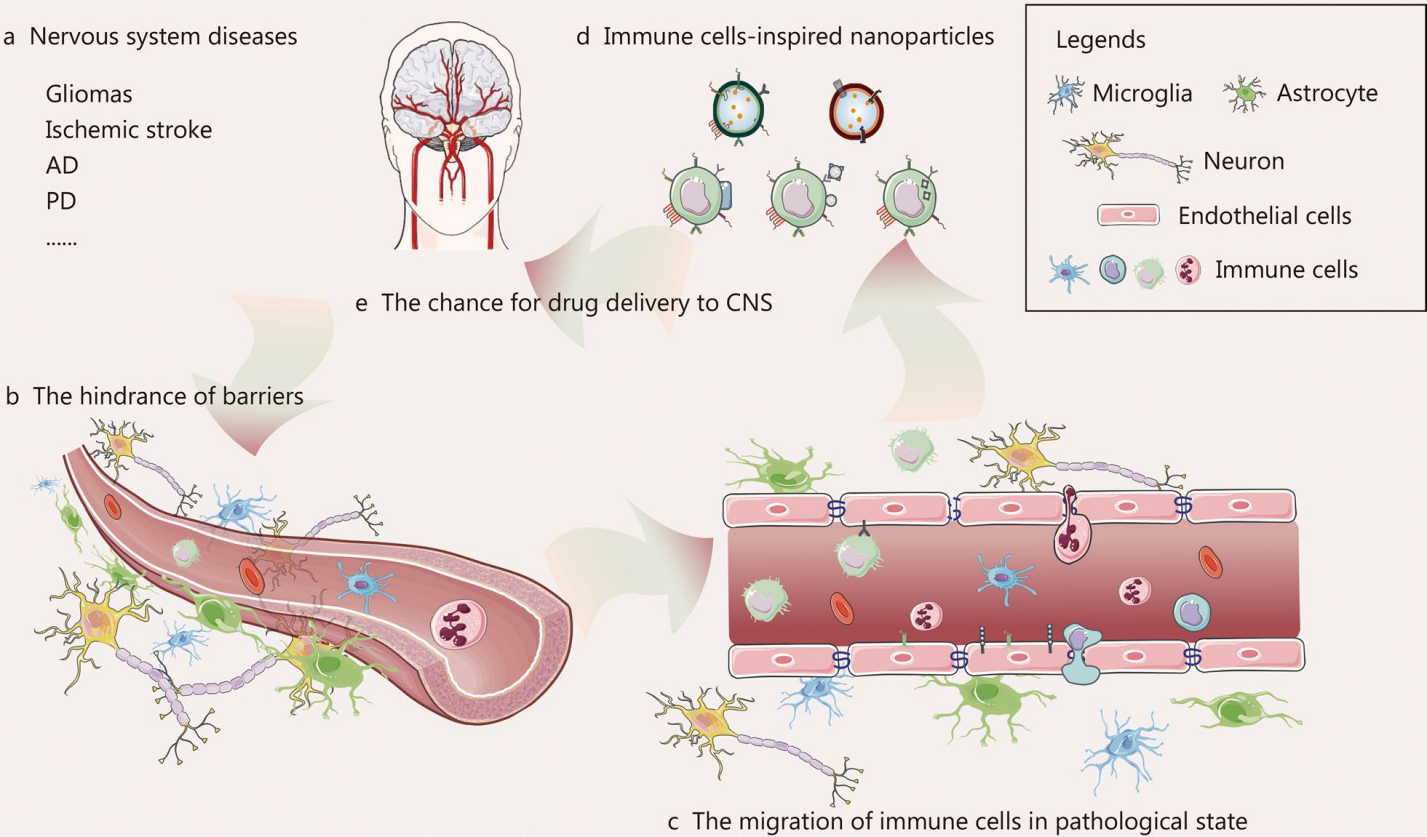
Graphical overview of the process toward immune cell-inspired nanoparticles for the treatment of central nervous system (CNS) diseases. **a** There are many nervous system diseases, including gliomas, ischemic stroke, Alzheimer’s disease (AD), Parkinson’s disease (PD), and several others. **b** The delivery of therapeutic drugs is hindered by barriers. **c** Peripheral immune cells migrate into CNS in the pathological state. **d** The migration of immune cells promotes the development of immune cell-inspired nanoparticles. **e** The immune cell-inspired nanoparticles provide a chance for drug delivery to the CNS

The advantages of drug delivery systems (DDS) in tissue and/or cell-targeted drug delivery, reduction of system cytotoxicity, prolongation of drug half-life, enhancement of dispersion, and biocompatibility of insoluble drugs have received increasing attention for several decades. In CNS diseases, many strategies based on nanoparticles have been developed to enhance the efficacy of delivering therapeutic drugs to the brain, including receptor-mediated transcytosis (utilizing specific ligands such as lactoferrin, transferrin, and certain antibodies), adsorptive-mediated transcytosis (employing cation nanoparticles), and carrier-mediated transport (modified with fatty acids, glucose, galactose, or mannose) [4, 5]. However, there remains much room for improvement (the incomplete specificity of nanoparticles, the toxicity of cation nanoparticles, the breakdown of the BBB, and so on). A deeper understanding of the microenvironment in CNS diseases, coupled with advancements in nanomedicine, has paved the way for bionic nanotherapeutics to emerge as a potential drug delivery strategy for the treatment of CNS disorders. Recently, the immune cells has been observed to migrate specifically to the brain parenchyma in CNS disease, which has sparked interest in developing immune cell-based nanoparticles for drug delivery to the CNS. The reemergence of intrinsic biological properties of the source cells, such as directed migration and the immune cell-based nanoparticles cloaking, offers the potential for effective drug delivery to the CNS (Fig. 1).

The objective of this article is to provide accessible insights into the relationship between immune cells capable of crossing brain barriers and the delivery of therapeutic drugs in the CNS. Following an overview of the anatomy, physiology, and pathology of the brain barrier, we investigate the role of peripheral immune cell involvement in CNS homeostasis and disease as well as mechanisms underlying interactions between immune cells and CNS. We subsequently explore the strategies for using immune cell-inspired DDS on CNS cells and tissues, along with their application in treating CNS diseases. Finally, from a neuroimmunology perspective, we examine current developments in immune cell-based DDS and their potential future regulation of the microenvironment associated with CNS disease. This review aims to summarize various aspects related to migration and roles played by immune cells across different diseases while highlighting promising approaches for leveraging these cells to deliver drugs to the CNS.

## The infiltration of immune cells into the brain

Enhanced understanding of the fundamental physiological mechanisms of the CNS is imperative for the advancement of immune cell-based CNS DDS. This section describes the physical structure of the CNS, including complex barrier systems responsible for maintaining homeostasis within this vital region, as well as the mechanisms through which immune cells migrate across the CNS.

### CNS structure

**Fig. 2:**
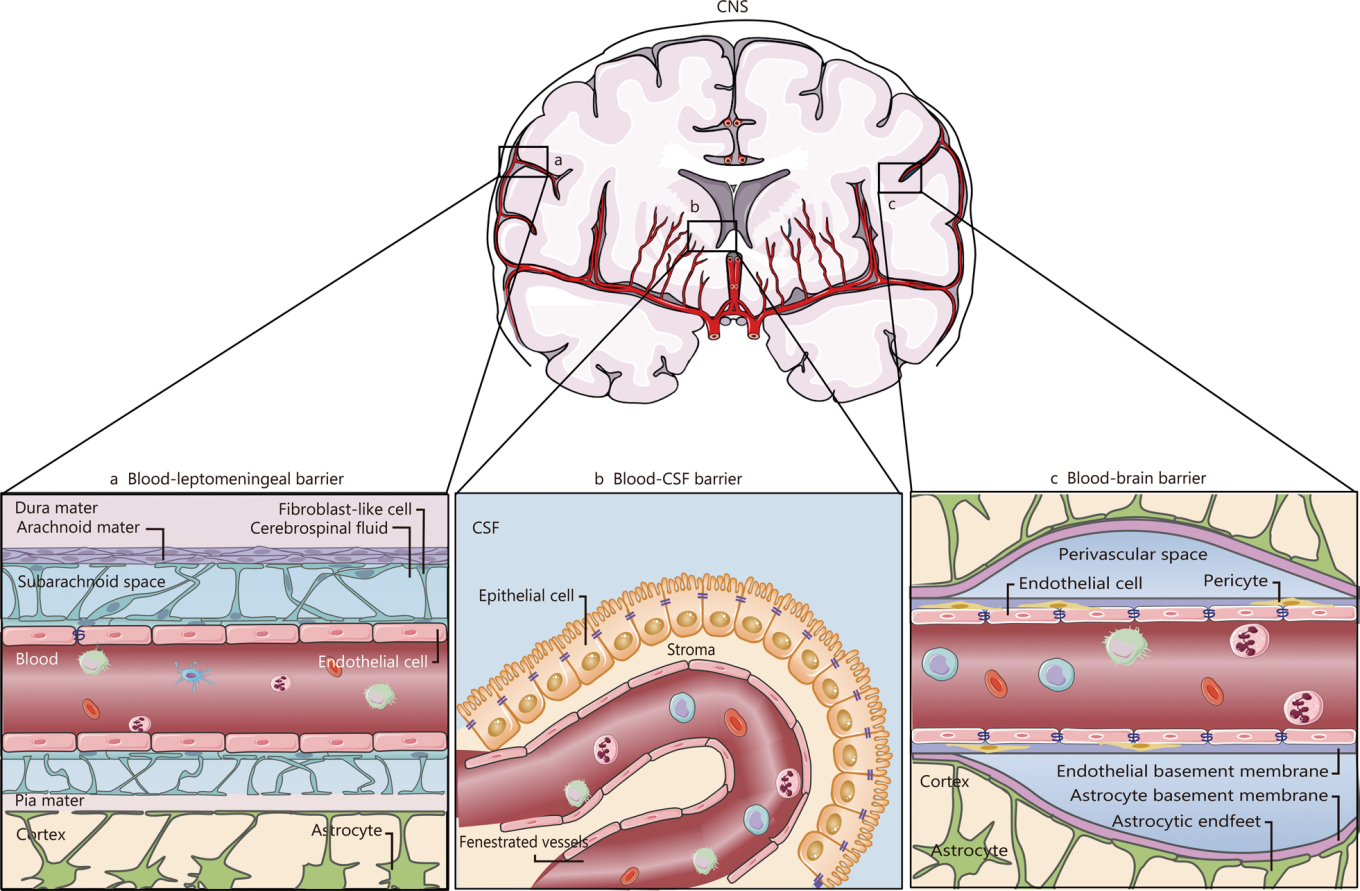
The structures of the blood-leptomeningeal barrier (**a**), blood-CSF barrier (**b**), and blood–brain barrier (**c)**. This figure is a modification of Fig. 1 by Mastorakos et al. [6]. BBB blood–brain barrier, CNS central nervous system, CSF cerebrospinal fluid

The CNS has traditionally been considered a site of immune privilege owing to its distinctive anatomical features. The meninges and cerebrospinal fluid (CSF) serve as protective barriers for the brain and spinal cord. There are three main physiological barriers between the peripheral blood and CNS, namely, the blood-leptomeningeal barrier, the blood-CSF barrier, and the BBB. These barriers play a crucial role in regulating the transportation of cells and molecules (Fig. 2). The meninges are anatomically divided into three layers: dura mater, arachnoid mater, and pia mater (Fig. 2a) [6, 7]. The dura mater contains lymphatics and fenestrated blood vessels without tight junctions (TJ), facilitating the entry of peripheral material and cells [6]. The arachnoid mater acts as an epithelial layer between the dura mater and the subarachnoid space. Its TJ and efflux pumps establish a barrier that separates the peripheral blood vessels of dura mater from CSF while filling the subarachnoid space [6]. The pia mater, which constitutes the innermost layer covering the surface of the brain and spinal cord, exhibits semipermeable to CSF allowing for soluble substances to pass through along with immune cells. This characteristic is crucial for CSF mixing with brain interstitial fluid, thereby exposing immune cells to CNS antigens [8]. The component of CSF is related to the choroid plexus, which is the primary site for CSF production. Like the dura mater, the vessels in the choroid plexus are fenestrated and without TJ. During homeostasis, the choroid plexus contains resident immune cells, including dendritic cells (DCs), macrophages, innate lymphoid cells, and CD4^+^ T cells, as well [7]. Similar to the arachnoid mater, ependymal cells surrounding the choroid plexus form a blood-CSF barrier between blood vessels and CSF (Fig. 2b), regulating the entry of immune cells and soluble factors into CSF through TJ [6]. Lastly, in CNS parenchyma, there exists a BBB that directly separates blood vessels from adjacent CNS tissue (Fig. 2c) [6]. The BBB is comprised of specialized endothelial cells, endothelial basement membrane, pericytes, astrocyte basement membrane, and astrocyte end feet. These specialized endothelial cells are tightly connected by claudins, occludins, annexin-1, and junction adhesives to form TJ, which are distinct from peripheral vessels. The BBB functions as the natural selective biochemical barrier between blood and CNS. It facilitates the transport of essential nutrients such as glucose, amino acids, vitamins, free fatty acids, minerals, and electrolytes from the bloodstream to the CNS by special transporters, maintaining CNS homeostasis by utilizing efflux pumps like p-glycoprotein to prevent harmful agents such as pathogens from entering the CNS. Within postcapillary microvessels, the endothelial basement membrane separates from the glial cell boundary, resulting in the formation of a perivascular space alongside a limited presence of antigen-presenting cells. The cerebral cortex exhibits a tight association between the endothelial basement membrane, glial cell boundaries, and specialized endothelial cells, with no presence of perivascular space. Under steady-state conditions, immune cells face challenges in accessing the brain parenchyma from the bloodstream due to the requirement of crossing both the vascular endothelium and glial junctions. Additionally, low expression levels of endothelial cell adhesion molecules further restrict peripheral immune cell entry into the CNS parenchyma. These inherent physiological barriers have evolved specialized mechanisms to ensure precise transmission of electrochemical signals within the CNS and maintain a balanced physical environment by regulating substance entry and exit. However, these tightly integrated barriers pose significant difficulties for drugs to reach the CNS.

### Interactions between the peripheral immune cells and CNS

The CNS is considered to be an immune-privileged organ, where the BBB, blood-CSF, and blood-leptomeningeal barriers tightly regulate the entry of immune cells into different compartments of the CNS. Under normal conditions, leukocytes such as granulocytes, T cells, and B cells remain within the blood vessels and typically do not penetrate healthy brain tissue. The primary immune defense during homeostasis is provided by innate immune cells, including parenchymal microglia and non-parenchymal macrophages [9].

In pathological states, the CNS microenvironment changes, leading to the production of pro-inflammatory cytokines, chemokines, and adhesion molecules, which facilitate the recruitment of circulating leukocytes across the BBB [10]. Concurrently, the BBB becomes disrupted, allowing peripheral immune cells to access the diseased brain [10]. There are three pathways through which immune cells can reach the CNS: CNS parenchymal blood vessels, leptomeningeal blood vessels, and the choroid plexus [11]. The migration of immune cells across the BBB is a complex process involving various molecules, including adhesion, activation, and migration proteins expressed on the BBB endothelial cells and/or migrating immune cells. Specifically, adhesion molecules such as intracellular adhesion molecule (ICAM)-1, ICAM-2, vascular cell adhesion molecule-1 (VCAM-1), and P-selectin which are expressed on endothelial cells, and their ligands like lymphocyte function-associated antigen-1 (LFA-1), very late antigen-4 (VLA-4), P-selectin glycoprotein ligand-1 (PSGL-1) play central roles in facilitating immune cell migration across the BBB. Additionally, chemokines such as C–C motif chemokine ligand (CCL) 2, CCL4, CCL5, and C-X-C motif chemokine ligand 12 (CXCL12) and their respective receptors CC-chemokine receptor (CCR) 1, CCR2, CCR5, and chemokine (C-X-C motif) receptor 4/7 (CXCR4/7) are also involved in the migration process [12].

**Fig. 3:**
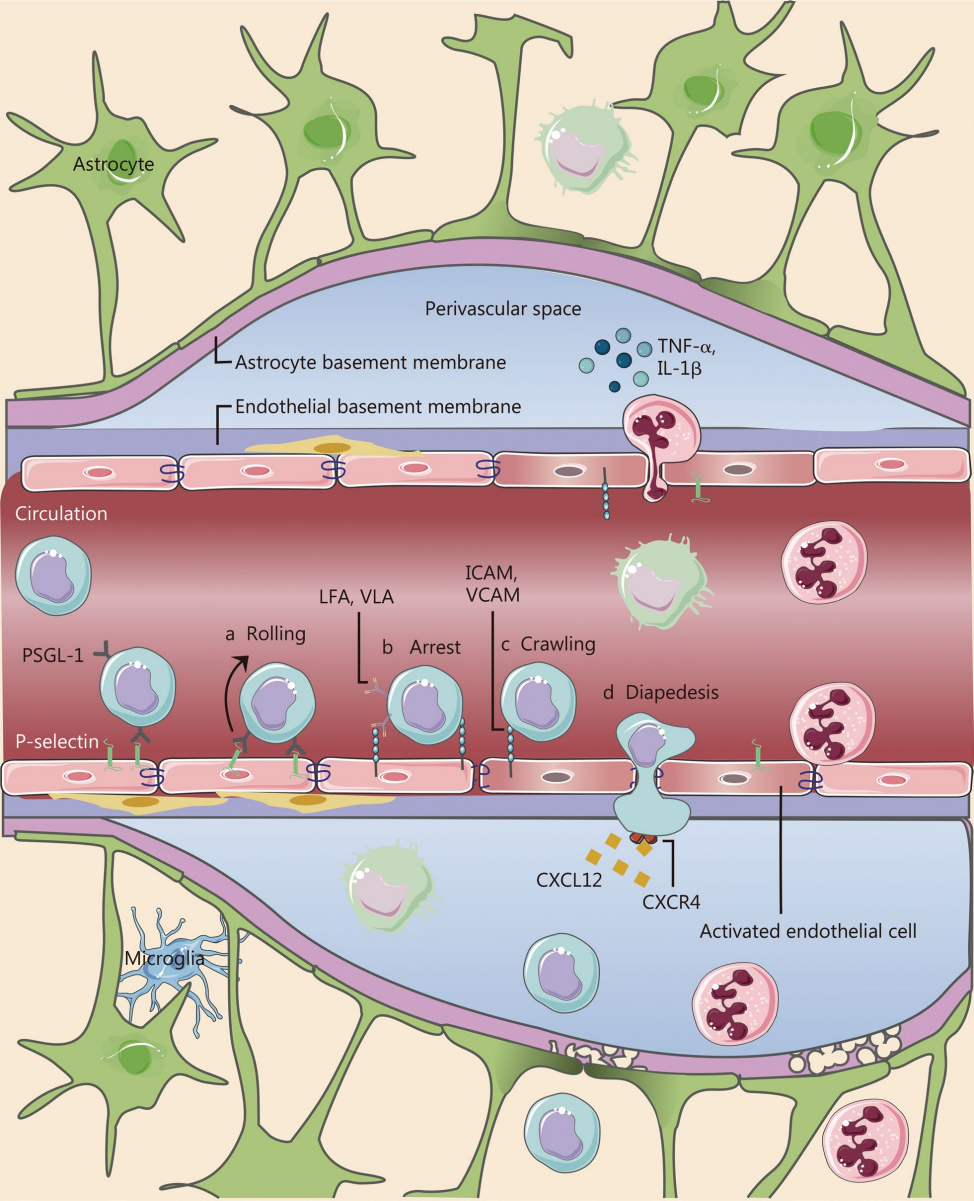
The process of immune cell migration across the blood–brain barrier (BBB) can be delineated as a series of consecutive stages. **a** Rolling. Immune cells engage with the endothelium through the interaction between PSGL-1 and P-selectin in the inflamed endothelial cells. **b** Arrest. The immune cells are activated upon transient contact with the endothelium, resulting in their firm adhesion to the endothelium. **c** Crawling. The immune cells polarize and crawl to locate permissive sites. **d** Diapedesis. The ultimate step is in which the immune cells traverse the barrier through either a paracellular (across the tight junctions) or transcellular pathway. This figure is a modification of Fig. 4 by Mastorakos et al. [6]. CXCL12 C-X-C motif chemokine ligand 12, CXCR4 chemokine (C-X-C motif) receptor 4, ICAM intracellular adhesion molecule, IL-1β interleukin-1β, LFA lymphocyte function-associated antigen, PSGL-1 P-selectin glycoprotein ligand-1, TNF-α tumor necrosis factor-α, VCAM vascular cell adhesion molecule, VLA very late antigen

The process of recruiting immune cells can be broken down into four distinct steps, as illustrated in Fig. 3 [13]. (1) The first step involves a process known as rolling, which serves to decrease the speed of immune cells to facilitate their recognition of proteoglycans expressed in endothelial cells [14]. The upregulation of E/P-selectin on endothelial cells facilitates interaction with PSGL-1 on immune cells, although this step is not essential for immune cell migration across the inflamed BBB. (2) The second step, arrest, is initiated by G-protein-coupled receptor signaling and involves the binding of integrins LFA-1, vVLA-4, and α4β1 integrin to their endothelial ligands, ICAM-1 and VCAM-1 [15]. (3) The third step, polarization and crawling, entails activated immune cells crawling against the flow direction on ICAM-1-coated surfaces to locate rare permissive sites for diapedesis across the BBB endothelium. (4) Finally, the fourth step, diapedesis, involves immune cells promptly crossing the endothelium through endothelial junctions, known as paracellular diapedesis, as well as transcellular immune cell diapedesis in the migration of immune cells across the BBB.

In a variety of CNS disorders, distinct brain microenvironments give rise to diverse molecular mechanisms for the attraction of peripheral immune cells to the CNS. For instance, the adhesion of neutrophils to endothelial cells is typically mediated by P-selectin, but in the context of cerebral ischemia, the involvement of PSGL-1 in the accumulation of neutrophils in the CNS is disregarded. Furthermore, the quantity and nature of the recruited peripheral immune cells are intricately linked to the specific diseases and will be detailed in the subsequent section.

## Immune-inspired nanoparticle delivery to the CNS

### The application of immune cell components in DDS

#### Cells

**Fig. 4:**
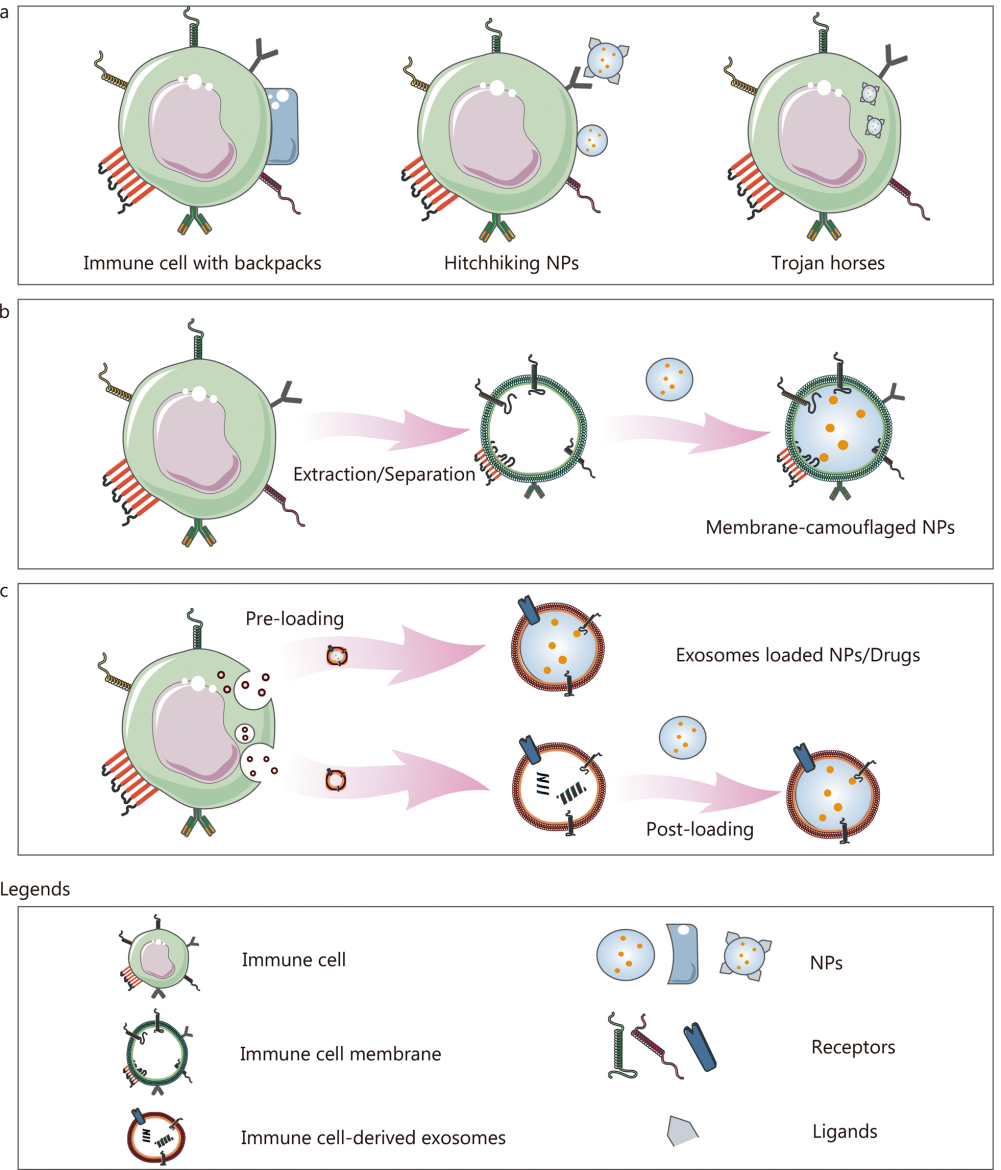
The utilization of immune cells as a delivery system for central nervous system drugs has been explored through various approaches. These include the direct use of immune cells as transport vehicles (**a**), the development of immune cell membrane-camouflaged nanoparticles (**b**), and the creation of exosome-loaded nanoparticles/drugs (**c**). NPs nanoparticles

Biological cells have been recognized as “living drugs” due to their inherent characteristics, such as strong biocompatibility and dynamic response to disease, including immunomodulation, tissue regeneration, and tumor destruction. Numerous cell therapy products, such as T cells, stem cells, and DCs, have been identified and approved for clinical use [16], with at least 1700 cell therapy products currently undergoing clinical trials [16]. Immune cell-based clinical trials account for over 60% of active cell therapy clinical trials. cells have been utilized as carriers to transport pharmaceutical cargo, such as drugs, mRNA, and peptides, to targeted sites, leveraging their natural capacities to target tissues, transport biological barriers, and reduce immunogenicity for mononuclear phagocyte system escape [17]. The mechanisms by which cells act as carriers include backpacks, hitchhiking, and Trojan horses (Fig. 4a) [18].

Depending on their physical characteristics, cellular backpacks can be categorized into two types: spherical structures and anisotropic shapes. Phagocytosis is a natural behavior of cells such as macrophages, neutrophils, and monocytes, and the geometry of particles plays a decisive role in their phagocytic fate. Anisotropically shaped particles can resist phagocytosis for longer periods compared to spherical structures [19]. Typically, cellular backpacks are polymer layers with diameters ranging from 7 to 10 μm and exhibit anisotropic shapes, enabling them to adhere to cells (Fig. 4a) [20–23]. The anisotropic shapes of cellular backpacks can hinder their cellular uptake by inhibiting the formation of actin structures necessary for phagocytosis, thereby decreasing the endosomal degradation of polymeric particles [19]. Preserving the biological functions of the cell-carriers, including their tropism, metabolic activities, and responsiveness to disease, is of paramount importance [24]. The assembled heterostructure is comprised of a cell-adhesive region, a payload region, and a release region [25]. It is important for the release region to be rapidly degraded under specific conditions, such as low pH and special temperatures [25]. Once the release region is degraded, the payload layers are exposed directly to the cellular environment, allowing the unloading of cargo such as drugs, proteins, or nanoparticles. The cell-adhesive region is responsible for anchoring the assembled heterostructure to the cell membrane, so it is essential to consider the surface features of the attached cells. Furthermore, it is imperative that the desirable cellular backpacks do not impact the functions of the attached cells, including their ability to migrate into the brain and their immunoregulation functions [20].

The term “hitchhiking nanoparticles” refers to the nanoparticles that directly attach to the membrane of carrier cells (Fig. 4a) [26, 27]. Similar to cellular backpacks, these hitchhiking nanoparticles are secured to the cell surface through receptor–ligand recognition, chemical bonding, or physical adhesion [27]. Different attachment methods offer distinct characteristics. For instance, hitchhiking nanoparticles attached to the cell-carriers through physical adhesion, such as electrostatic interactions, hydrophobic interaction, van der Waals forces, and hydrogen bonding [28–31], require minimal modification. While the weak interaction between hitchhiking nanoparticles and cell-carriers results in limited anchoring stability in circulation, the abundance of receptors on the cell-carriers provides a reliable, reproducible, and straightforward attachment method for the nanoparticles. Moreover, by modifying the ligands on the hitchhiking nanoparticles, multiple cells can be used as potential carriers. However, the numerous receptors on various cells can lead to non-specific attachment [17]. Additionally, the interaction between receptor-ligand may disrupt the biological functions of the cell-carriers [27]. The plentiful proteins on the cell surface offer numerous active groups, such as amines and thiols, for hitchhiking nanoparticles [32, 33]. Furthermore, non-natural functional groups on the cell surface, achieved through metabolic strategies (azide moieties) or chemically generated reactive groups (e.g., aldehydes), can also covalently link nanoparticles. Amino groups react with biotin moieties, which can facilitate noncovalent cell surface conjugation mediated by (strept) avidin. Some literature shows that amino groups on the cell surface can directly covalently conjugate isocyanate, *N*-hydroxysuccinimide, cyanuric chloride, or succinimidyl functionalized nanoparticles [34–36]. The cysteine thiol groups on the cell surface serve as anchors for nanoparticles via maleimide-thiol coupling (e.g., maleimide-functioned liposomes) and disulfide bond (e.g., pyridyldithiopropionate-functionalized liposomes) [37]. The reversible nature of the disulfide bond allows for the detachment of nanoparticles in high concentrations of glutathione. Non-natural aldehyde groups modified on the cell surface can be linked to amine-functionalized dendrimers or quantum dots through Schiff base formation. Metabolically incorporated azide moieties on the cell surface can anchor cyclooctyne-functionalized polyamide-amine dendrimers through strain-promoted azide-alkyne cycloaddition [38]. The strongest interaction provided by chemical bonding ensures stable attachment between hitchhiking nanoparticles and cell-carrier, thus limiting detachment and uptake of the nanoparticles in non-target tissues [33]. It is important to note that chemical reactions and group modifications on the cell surface should not affect cell viability and motility.

The Trojan horse strategy relies on the natural process of cell-carriers engulfing drug-loaded nanoparticles, highlighting the phagocytic capability of cell-carriers [39]. Naturally, macrophages or monocytes are the preferred candidates due to their superior phagocytosis [40]. The phagocytosis of macrophages for the nanoparticles involves specific receptor interaction, rearrangement of cellular cytoskeleton in the cytosol, participation in actin polymerization, and formulation of phagosome [41]. Nanoparticle properties such as size, shape, surface chemistry, and mechanical properties influence macrophage phagocytosis. For example, hydrophobic nanoparticles are more easily engulfed by macrophages compared to hydrophilic ones, and cationic nanoparticles are more susceptible to phagocytosis than anionic ones due to the negative charge of the macrophage cell membrane.

To enhance the number of nanoparticles absorbed by cell-carriers, various methods are used, including physical techniques like hypoosmotic hemolysis and electroporation, chemical disruption, cell‑penetrating peptides, and liposome fusion. It is crucial to prevent the efflux of internalized nanoparticles to improve the loading efficiency of cell-carriers. Once inside the cell-carriers, nanoparticles are shielded from interacting with non-target tissue in vivo. However, there is a risk of the cell-carriers degrading the internalized nanoparticles, which can lead to inaccurate targeting. Furthermore, the cytotoxicity and release behavior of internalized nanoparticles, such as those loaded with anti-cancer drugs, can significantly impact their therapeutic effectiveness. The Trojan horse strategy has been successful in overcoming barriers in various CNS disease models, including acute neuroinflammation, PD, depression, glioma, and HIV-1 encephalitis.

#### Cell membranes

Cell membranes are comprised of organized lipids, polysaccharides, and proteins, and play important roles in communication with the extracellular microenvironment, including adhesion, migration, recruitment, cell–cell interaction, signal transduction, and material transport [42]. Extracted cell membranes, which contain molecular machinery like CD47, help maintain important functions such as immune evasion, prolonged circulation, transport across the BBB, and targeting of pathological sites [43]. The potential application of natural functions derived from cell membranes has sparked considerable research interest in cell membrane-camouflaged nanoparticles. The diverse sources of cell membranes, including red blood cells [44], platelets [45], stem cells [46], immune cells [47–49], tumor cells [50], bacteria [51], and engineered cells, expand the potential application of these nanoparticles due to their specific biological function.

The process of creating nanoparticles camouflaged with cell membranes involves two main steps: extracting and collecting the cell membrane, and forming vesicles (Fig. 4b) [52]. The extraction of the cell membrane is essential for preserving the complete characteristics of the cell membrane [53]. Currently, cell membrane extraction methods include mild lysis (such as hypotonic lysis and freeze–thaw cycles), chemical lysis (using lysis buffers, detergents, pH, salts, and enzymes), and mechanical lysis (involving homogenization, sonication, and microfluidic electroporation) [53]. Hypotonic lysis takes advantage of the differences in concentration between the hypotonic solution and the cytoplasm, facilitating the continuous diffusion of water molecules into the cell, and causing cell swelling and lysis. Freeze–thaw cycles break the cell walls as ice crystals form and contract during multiple cycles. These methods are relatively gentle, and broken cells are usually further disrupted through homogenization. Chemical lysis uses a lysis buffer to change the pH and rupture the cell membrane. Additionally, the addition of detergents in lysis buffers can solubilize membrane proteins, disrupting the cell membrane. Sonication involves using sound energy to sound energy to agitate cells and their components, leading to cellular disruption and lysis. The sonication process can completely split cells into an upper liquid containing crude cytoplasm extract, but the high sound energy can cause protein denaturation, so maintaining a low temperature with ice-cold lysis buffers is crucial. Regardless of the method used to extract cell membranes, it should be done in a gentle environment to minimize protein degeneration on the cell membrane.

Subsequently, the collected cell membranes are predominantly enveloped around the inner core nanoparticles through co-extrusion and sonication (Fig. 4b). Co-extrusion is a commonly employed technique in the fabrication of cell membrane-camouflaged nanoparticles. In this process, the blend of inner nanoparticles and the collected membrane is continuously extruded through—porous membranes of varying sizes to achieve vesicle-particle fusion. While co-extrusion is effective and provides stability for creating multi-layer and multi-functional structures, it is not suitable for large-scale production. Sonication, on the other hand, utilizes ultrasonic waves to facilitate the fusion of co-incubated cell membranes and nanoparticles. The optimization of ultrasonic parameters (power, frequency, and time) is essential for improving fusion efficiency and minimizing drug leakage and protein denaturation. Furthermore, the microfluidic electroporation method is used to fuse cell membranes and nanoparticles [54]. This method offers notable advantages in preserving the integrity of the cell membranes and reducing the loss of cell surface proteins. Specifically, the system consists of two channels for mixing, an outlet, and an electroporation segment that delivers electric pulses). When the mixture flows into the electroporation segment, the electric pulses induce the formation of the transient pores in the plasma membranes, facilitating the entry of inner nanoparticles [54].

Additionally, to enhance the capabilities of cell membrane-camouflaged nanoparticles beyond the inherent function of natural cells, the membranes were altered based on the physicochemical properties of cell membranes. This involved modifications such as targeted peptides [55, 56], and cell-penetrating peptides [57]. Alternatively, transfection or transduction with non-viral or viral vectors has been employed [58, 59]. However, the intricate and laborious preparation processes restrict membrane yield and availability. Moreover, it is imperative to explore the approach for establishing the correct orientation of the membrane coating on the nanoparticles, as this is closely related to the biological function of cell membrane-camouflaged nanoparticles.

#### Exosomes

Extracellular vehicles (EVs) are a diverse group of membrane particles released by cells, including exosomes, microvesicles, and apoptotic bodies, each with distinct biogenesis processes [60, 61]. Microvesicles and apoptotic bodies are formed through outward budding and fission of the cell membrane [62, 63], while exosomes, ranging in size from 30 to 150 nm, are secreted via the endosomal pathway [64]. In the context of DDS, EVs generally refer to exosomes.

The formation of exosomes includes three stages: endosome, intra-luminal vesicles, and multivesicular body [63]. During this process, various biomacromolecules, such as RNA, DNA, and proteins, are enclosed within the lumen or lipid bilayer, contributing to the diverse functions of exosomes, including mediating cell–cell signal communication, transporting bioactive molecules, and aiding in the immune response [65]. Exosomes are spherical lipid-bilayered particles with an amphiphilic lipid bilayer and an aqueous core, resembling liposomes. The unique biological features and specific structure of exosomes have led to their application in DDS. When used for drug delivery, it is crucial to preserve the vesicular structure and proteins during exosome isolation, making mild and efficient isolation and purification methods essential due to the complexity of bodily fluids or cell culture supernatants. According to the Minimal Information for Studies of Extracellular Vesicles 2018 Guidelines, the isolated extracellular exosomes must exhibit at least three positive protein markers (transmembrane/lipid-bound protein, cytosolic protein, and negative protein marker) [66].

Currently, various methods are employed for the isolation and purification of exosomes, including ultracentrifugation, ultrafiltration, filtration, immune-affinity-based isolation, precipitation isolation methods, and microfluidics-based isolation. These methods are selected based on the distinct characteristics of exosomes, such as density, shape, size, and surface proteins [67]. Ultracentrifugation, which encompasses both ultracentrifugation and density gradient ultracentrifugation, is the most commonly used technique. Differential ultracentrifugation involves the use of different centrifugal speeds and times to separate exosomes, while density gradient ultracentrifugation utilizes density gradient mediums to separate exosomes through the action of gravity or centrifugal force field, with iodixanol and sucrose being commonly used as the density gradient medium [68–70]. Although the ultracentrifugation method can yield high-purity exosomes with high efficiency, the sustained high centrifugal force may lead to the rupture of the exosome membrane or the loss of active exosomes and their components during the separation process. Filtration, which includes ultrafiltration and filtration dialysis, is a size-based separation technology that can isolate exosomes more quickly than ultracentrifugation without requiring additional equipment [71]. However, the efficiency of separation is limited by the clogging and trapping of pores by vesicles, leading to less purity of isolated exosomes. The immuno-affinity-based isolation method utilizes specific proteins in the membranes of exosomes, such as tetraspanins, epithelial cell adhesion molecule, and Rab-5B, to interact with antibodies in the column that attach to specific exosome surface ligands [72, 73]. For example, the magnetic beads enrichment method employs magnetic nanoparticles attached to antibodies targeting exosomal biomarkers to separate exosomes with high efficiency and low cost [64]. The advantages and disadvantages of different isolation methods are summarized in Table 1 [74].

**Table 1:** The comparison of methods for exosome isolation

Isolation method	Advantages	Disadvantages
Differential ultracentrifugation	Low cost Low contamination risk with extra isolation reagents Suitable for large volume preparation	High equipment requirement Time consuming Labor intensive Potential mechanical damage due to high-speed centrifugation Protein aggregation Not suitable for small volume diagnosis Low portability
Density gradient ultracentrifugation	High purity of products Allowing separation of the subpopulation of exosomes	Lower volume processability High equipment requirement Time consuming Labor intensive Potential mechanical damage due to high-speed centrifugation Not suitable for small volume diagnosis Low portability
Ultrafiltration	Low equipment cost Fast procedure Good portability	Moderate purity Potential deterioration induced by shear stress Possible loss due to clogging and membrane trapping
Immuno-affinity-based isolation method	Suitable for separating exosomes of specific origin High-purity exosomes Easy to use No chemical contamination	High-cost antibodies Exosome markers must be optimized Low processing volume and yields Extra step for exosome elution may damage native exosome structure

Exosomes, with their distinctive structure comprising an aqueous core and a lipid bilayer, have the capability to encapsulate both hydrophilic drugs and hydrophobic molecules via pre-loading or post-loading methods (Fig. 4c). Pre-loading can be achieved through intracellular expression of biological cargos, such as peptides and proteins, via cell transfection, or by uptake of therapeutic molecules into the originating cells by pre-incubation [75]. These cargos and drugs can be integrated into exosomes during the biogenesis process [75]. In addition, post-loading involves a simple and direct loading method through incubation, depending on passive diffusion to incorporate the target drug into the exosomes [67, 76]. The efficiency of loading is contingent upon drug characteristics such as solubility and pH and is constrained by the cellular tolerance of the dose. Furthermore, the separation of unloaded drugs poses a hindrance to its application. To enhance drug loading efficiency, mechanical or chemical techniques such as sonication, extrusions, electroporation, freeze–thaw cycles, and permeabilization (Saponin) are used to open the exosome membranes, allowing for increased drug diffusion into the purified exosomes [77]. While these technologies indeed improve drug loading efficiency, they may compromise the structural integrity of the exosome membrane, potentially leading to drug leakage in vivo. Meanwhile, compared to pre-loading and incubation methods, the complex technologies involved in the post-loading method lead to the introduction of more uncontrollable factors in the construction process.

Exosomes sourced from immune cells and stem cells, among others, are applied in CNS diseases [78, 79]. The exosomes derived from different cells exhibit distinct distribution characteristics in the brains of mice due to variations in the molecules expressed on their surface. To identify superior carriers for treating neurodegenerative disorders, the physicochemical properties, ability to cross the BBB, and accumulation in neuronal cells of exosomes from three types of origin cells, including macrophages (mEVs), neurons (nEVs), and astrocytes (aEVs), were investigated. Among these, the brain accumulation levels of mEVs in a transgenic mouse model of PD were significantly higher than those of nEVs or aEVs, potentially attributed to the highest levels of tetraspanins and integrins in mEVs compared to nEVs and aEVs [80]. Consequently, mEVs were proposed as the most promising nanocarrier system for drug delivery to the brain [80, 81]. However, there are challenges to be addressed for the future application of exosomes in the clinic, including the standardization of isolation techniques with low-cost and efficient drug-loading methodologies.

### Immune-inspired nanoparticles in CNS disease

#### Glioma

**Fig. 5:**
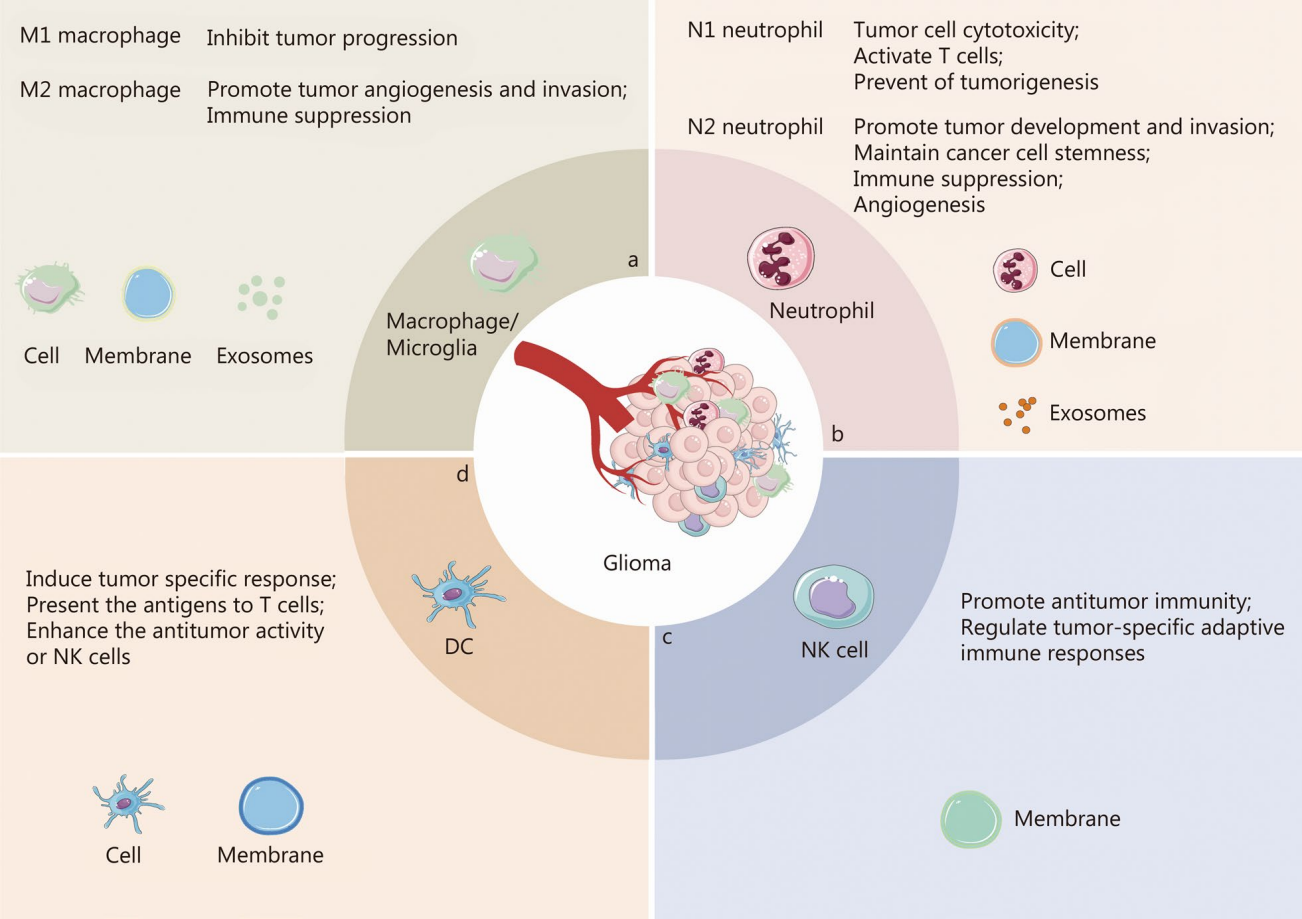
The involvement of immune cells in glioma and their roles as well as potential applications in immune cell-based drug delivery systems (DDS). This includes macrophage and microglia (**a**), neutrophil (**b**), natural killer (NK) cell (**c**), and dendritic cell (DC, **d**)

Gliomas represent the most prevalent primary malignant tumors of the CNS. While surgical resection is a standard therapeutic approach, complete removal of tumor cells infiltrating the normal brain tissue is unattainable. Consequently, residual tumor cells are typically managed with pharmacotherapy. However, the BBB or the blood–brain tumor barrier hinders the effective delivery of chemotherapy agents, leading to tumor recurrence. The glioma microenvironment significantly influences tumor initiation and progression. Immunohistochemical analysis of human gliomas has revealed substantial infiltration of immune cells [82]. Tumor cells, endothelial cells, immune cells, and various cytokines collectively constitute the glioma tumor microenvironment (TME). Infiltrating immune cells, such as macrophages, microglia, neutrophils, regulatory T cells (Tregs), myeloid-derived suppressor cells, T lymphocytes, natural killer (NK) cells, DCs, etc., play an important role in regulating immune responses within the microenvironment (Fig. 5) [83–85] and have spurred the development of immune cell-related biomimetic DDS (Table 2) [86–112].

**Table 2:** Application of immune cell-based DDS in glioma

Immune cells	Components	Loaded drugs/nanoparticles	Methods	Model in vivo	Functions	References
Monocyte	Cell	Conjugated polymer nanoparticles	Trojan horse	GL261 orthotopic gliomas mice model	Target BBB and tumor Increase the PDT treatment efficacy	[86]
Monocyte	Cell	Nano-DOX	Trojan horse	Orthotopic GBM xenografts mice model	Target BBB and tumor cells Induce tumor cell damage by promoting DAMP emission	[87]
Macrophage	Cell	Photothermal nanoprobe (MFe_3_O_4_-Cy5.5)	Trojan horse	C6 orthotopic gliomas rat model	Target BBB and tumor, and penetrate deeply Perform fluorescence, photoacoustic, and magnetic resonance imaging Induce local photothermal therapy Inhibit the recurrence of glioma after surgery	[88]
Macrophage	Cell	C6/Dir loaded nanoparticles	Trojan horse	U87 orthotopic gliomas mice model	Target BBB and tumor Promote deeper permeation in tumor tissue	[89]
Macrophage	Cell	Dox	Trojan horse	–	Enhance drug release efficacy from macrophage by photochemical internalization	[90]
Macrophage	Cell	β-Glucans prodrug nanoparticles	Trojan horse	ALTS1C1 and LCPN orthotopic gliomas mice model	Target BBB and tumor cells Glutathione responsive drug release Suppress tumor growth and prolong survival time	[91]
M2 macrophage	Cell	Nano-DOX	Trojan horse	Orthotopic GBM xenografts mice model	Target BBB and tumor Modulate tumor immune microenvironment Suppress tumor growth	[92]
M1 macrophage	Cell	DOX-loaded PLGA nanoparticles	Trojan horse	U87 intracranial glioma-bearing mice model	Target BBB and tumor cells Induce tumor cell apoptosis Suppress tumor growth and prolong survival time	[93]
Macrophage	Membrane	MnO_2_ and cisplatin-loaded redox-responsive poly(N-vinylcaprolactam) nanogels	Hypotonic lysis/freeze–thaw/centrifugation Co-extrusion	C6 orthotopic brain glioma model	Target BBB Deplete excess glutathione Exhibit magnetic resonance imaging-guided chemotherapy/chemodynamic therapy	[94]
Macrophage	Membrane	DSPE-PEG-loaded IR-792 nanoparticles	Lysis buffer, sonication, and centrifugation Co-extrusion	U87L orthotopic GBM model	Target BBB and tumor Suppress tumor growth and prolong survival time	[95]
PD-1 overexpressed macrophage	Membrane	RAPA-loaded PLGA nanoparticles	Homogenization and centrifugation Co-extrusion	C6-Luc orthotopic gliomas mice model	Target BBB and the tumor microenvironment Block the immune checkpoint Activate the CD8^+^ CTL	[96]
M1-macrophage	Extracellular vesicles	CPPO and Ce6 modified and hypoxia-activated prodrug AQ4N	Incubation, ultracentrifugation, incubation and ultrafiltration (Pre-and post-loading)	Orthotopic gliomas mice model	Target BBB and tumor cells Modulate the immunosuppressive tumor microenvironment The chemiexcited photodynamic therapy and hypoxia-activated chemotherapy	[97]
Macrophage	Exosomes	SPIONs) and Cur	Differential centrifugation and ultrafiltration; Electroporation (post-loading)	U251 orthotopic gliomas mice model	Target BBB and tumor	[98]
Functionalized macrophage	Exosomes	Panobinostat and PPM1D-siRNA loaded positively charged micelles	Centrifugation and ultrafiltration; Hypotonic lysis Co-extrusion	Orthotopic DIPG-bearing mice	Target BBB and tumor Suppress tumor growth and prolong survival time	[99]
Microglia	Cell	PTX loaded liposome	Trojan horse	U87 MG and GL261 orthotopic gliomas mice model	Target BBB and tumor Transport drug to glioma cells via extracellular vesicles and tunneling nanotubes Suppress tumor progression	[100]
Microglia	Membrane	ZOL loaded nanoparticle	Mechanical crushing; Co-extrusion	GL261/TR orthotopic gliomas mice model	Target BBB and tumor cells Inducing apoptosis and inhibiting the migration and invasion of temozolomide-resistant GBM cells Improve the immunosuppressive and hypoxic microenvironment	[101]
Microglia	Membrane	Fe_3_O_4_-siPD-L1	Hypotonic lysis/freeze–thaw/centrifugation; Sonication and co-exclusion	Luc-GL261/TR orthotopic gliomas mice model	Target BBB and tumor cells Increase effector T cells and M1-type microglia Induce ferroptosis of GBM cells and maturation of DC cell Suppress tumor growth and prolong survival time	[102]
Neutrophil	Cell	PTX loaded cationic liposome	Trojan horse	Glioma surgical resection model	Target inflamed regions in brain and tumor cells Inflammatory responsive drug release Suppress the recurrence and improve survival rates	[103]
Neutrophil	Cell	DOX-loaded magnetic mesoporous silica nanoparticles	Trojan horse	Glioma surgical resection model	Target inflamed regions in brain and tumor cells Suppress tumor growth and prolong survival time Promote tumor cells and TAM apoptosis	[104]
Neutrophil	Cell	Escherichia coli membrane-enveloped, PTX-loaded magnetic nanogels	Trojan horse	Glioma surgical resection model	Target inflamed regions in brain and tumor cells Inhibit the proliferation of tumor cells	[105]
Neutrophil	Cell	PTX and anti-PD-1 antibody-loaded nanosensitizer	Trojan horse	GL261 tumor-bearing C57BL/6 mice	Target inflamed regions in brain and tumor cells Ultrasound responsive drug release Kill tumor and induce local inflammation Improve survival rates	[106]
Neutrophil	Exosomes	DOX	Centrifugal and filter separation, Sonication, and incubation (post-loading)	Zebrafish and C6-Luc glioma-bearing mice models	Target BBB and tumor cells Suppress tumor growth and prolong survival time	[107]
Neutrophil/ Macrophage	Membrane	RAPA-loaded PLGA nanoparticles	Hypotonic lysis and homogenizer; Sonication and co-extrusion	C6-Luc glioma-bearing mice models	Target inflamed regions in brain and tumor cells Eliminating glioma cells and inducing durable tumor regression	[108]
NK cell	Membrane	AIE nanoparticles	Lysis buffer/differential ultracentrifugation and co-extrusion	Orthotopic glioma models	Target BBB and tumor cells Trigger TJs disruption and actin cytoskeleton reorganization Suppress tumor growth	[109]
DC cell	Cell	Nano-DOX	Trojan horse	Orthotopic human GBM xenografts mice model	Target BBB and tumor Enhance DC-driven anti-GBM immune response	[110]
DC cell	membrane	RAPA-loaded PLGA nanoparticles	Lysis buffer and centrifugation Co-extrusion	C6-Luc glioma C57 mice	Target BBB and tumor Enhance immune response Suppress tumor growth and prolong survival time	[111]
DC cell	C6 and DC membrane	DTX nanosuspensions	Hypotoniclysis/freeze–thaw/differential ultracentrifugation and sonication	Intracranial glioma- mouse model	Target BBB and tumor Enhance DTX anti-tumor efficiency Activate downstream immune system	[112]

*AIE* aggregation-induced emission, *BBB* blood–brain barrier, CPPO bis(2,4,5-trichloro-6-carbopentoxyphenyl) oxalate, *CTL* cytotoxic T-lymphocyte, *Cur* curcumin, *DAMP* damage-associated molecular patterns, *DC* dendritic cell, *DIPG* diffuse intrinsic pontine glioma, *Dir* 1,1′-dioctadecyl-3,3,3′,3′-tetramethyl indotricarbocyanine Iodide, *DOX* doxorubicin, DSPE-PEG 1,2-Distearoyl-sn-glycero-3-phosphoethanolamine-*N*-[methoxy(polyethyleneglycol)-2000], *DTX* docetaxel, *C6-Luc* C6 glioma cells that luciferase reporter-gene labeled, *GBM* glioblastoma, *PD-1* programmed cell death-1, *PDT* photodynamic therapy, *PLGA* poly(lactic-*co*-glycolic acid), *PPM1D* protein phosphatase 1, *PTX* paclitaxel, *RAPA* Rapamycin, *SPIONs* superparamagnetic iron oxide nanoparticles, *TAM* tumor-associated macrophages, *TJs* tight junctions, *ZOL* Zoledronate. “–” represent no mentioned

##### Macrophage

An abundance of tumor-associated macrophages (TAMs), including macrophages, monocytes, and microglia, are observed in the gliomas of experimental animals and patients’ biopsies. Microglia and macrophages have distinct cellular origins. Microglia originates from immature yolk sac progenitors and expresses CCR2^−^, CX3CR1^high^, CD11b^+^, F4/80^+^, and CD45^low^ markers. While monocytes are generated from hematopoietic stem cells that differentiate into granulocyte macrophage progenitors, and then into monocyte-DC progenitors. Subsequently, mature Ly6C^high^, CCR2^+^ CX3CR1^low/int^ inflammatory monocytes are released into circulation to colonize peripheral organs under both normal and inflammatory conditions. In gliomas, these monocytes infiltrate into the CNS and differentiate into TAMs, expressing CX3CR1, CCR2, CD45^high^, F4/80^+^, and CD11b^+^ [113].

In the 1990s, researchers found that exogenous macrophages would migrate toward inflammatory body regions or reticuloendothelial organs for elimination [114]. Additionally, a study suggested that the migratory capacity of paramagnetic nanoparticles ingested by monocytes to cross a brain endothelial monolayer was unaffected [115]. Subsequently, Valable et al. [116] demonstrated that intravenously injected micrometer-sized particles of iron-oxide-labeled Mo/Ma could target a brain tumor by magnetic resonance imaging tracking in vivo, leading to the development of TAMs as carriers of drugs, nanoparticles, or photosensitizers to target tumors and tissues surrounding tumor by the Trojan horse strategy [86–93]. The uptake capacity of TAMs is critical to drug delivery, determined by the surface properties and morphology of nanoparticles (cargos) [117, 118]. For example, the uptake efficiency of bare gold–silica nanoshells by macrophages was 4 times that of PEGylated gold–silica nanoshells [117]. Furthermore, gold–silica nanorods were more likely to be ingested by macrophages than gold–silica nanoshells. Moreover, the uptake efficiency of macrophages was associated with its phenotype [86]. It is crucial to improve therapeutic effects on glioma through the promotion of drug offloading from macrophage carriers and the absorption of drugs by tumor cells after the migration of TAM carriers into CNS employing the driver of inflammatory cytokines [89, 90].

Utilizing the Trojan horse strategy, the nanoparticles can enter the brain. Miao et al. [91] prepared a prodrug by attaching an anticancer drug (temozolomide) to β-glucans through a disulfide-containing linker. The self-assembled nanoparticles from this prodrug specifically target intestinal microfold cells due to the connection between β-glucans and the membrane phagocytic pattern-recognition receptor Dectin-1 on intestinal microfold cells in intestinal Peyer’s patches. These nanoparticles are engulfed by local macrophages in Peyer’s patches. Taking advantage of the tumor-homing ability of macrophages, which is driven by various chemoattractants secreted by the tumor cells, the macrophage-hitchhiked prodrug enters the circulatory system via the lymphatic system and can transverse the BBB to accumulate in the tumor. Additionally, the overexpressed glutathione can specifically break down the prodrug nanoparticles, resulting in the release of conjugated temozolomide [91].

Considerable research efforts have been directed toward investigating the potential of TAMs as active carriers capable of bypassing the BBB to deliver chemotherapy drugs for the treatment of glioblastoma. However, the impact of exogenous TAM carriers that migrate into tumors in the brain remains uncertain. Li et al. [92] have developed TAMs loaded with Nano-Doxorubicin (DOX), which not only have the ability to infiltrate tumors but also can reprogram the exogenous TAM carriers from a pro-tumor phenotype (M2) to an anti-tumor phenotype (M1), thereby suppressing tumor growth. Specifically, the released Nano-DOX from TAMs induces damage-associated molecular patterns, which in turn promote the recruitment of Nano-DOX-TAMs and TAMs. In addition, the study involving M1 macrophage-carried DOX-loaded PLGA nanoparticles in the U87 glioma model has confirmed the potential efficacy of M1 macrophage carriers, including their tumor-homing properties and their ability to induce cellular apoptosis [93].

In comparison to macrophages, macrophage-derived membranes or exosomes cannot transition into an immunosuppressive TAM phenotype induced by the TME. However, the macrophage-derived exosomes retain the inherent ability to penetrate the BBB [94–97]. More importantly, exosomes derived from M1 macrophage- can alter the immunosuppressive TME via M2-to-M1 polarization [97]. To enhance BBB penetration, ultrasound exposure has been used [119]. It has been reported that either macrophage-derived exosomes or blood serum-derived exosomes can traverse BBB models and accumulate in glioma cells [97]. Meanwhile, the phospholipid bilayers of exosomes’ membrane ensured the feasibility of constructing tumor cell-targeted exosomes by incubating them with the ligands [98, 99]. Additionally, genetic engineering can confer additional functions to macrophage-derived membranes or exosomes, such as immune checkpoint blockade by overexpression of PD-1 [96].

##### Microglia

Microglia, the resident macrophages in the CNS, can be distinguished from bone marrow-derived CD45^+^ macrophages by their CD45 expression [120]. In the glioma microenvironment, microglia can be recruited by gliomas through chemoattractants such as CCL5 [120], CCL2 [121], CX3CL1 [122], and CXCL12 [123], and can penetrate the tumor. These characteristics make microglia suitable candidates for delivering drugs for glioma treatment [100–102]. Du et al. [100] developed a liposome-carrying microglia to deliver paclitaxel (PTX) for glioma treatment. The engineered microglia could migrate towards glioma cells across the BBB and penetrate the tumor, as evidenced by stronger fluorescence intensity in the brain of the orthotopic glioma mouse model and the presence of fluorescence in deeper regions of tumor spheroids. Additionally, they observed that the transfer of loaded nanoparticles from the engineered microglia to glioma cells was mediated by the formation of EVs and tunneling nanotubes, overcoming the limitations of traditional nanoparticle delivery systems [100].

The impact of microglia on the development of gliomas is significant and should be considered in the design of microglia-based DDS. Research has shown that the endogenous microglia in the TME can be reprogrammed to promote tumor growth through the transfer of extracellular miR-21 released by glioma cells [124]. However, the role of transmigrated exogenous microglia in glioma development is not well understood. Microglia in the TME plays a central role in brain tumor pathobiology, as they secrete factors such as stress-inducible protein, epidermal growth factor, transforming growth factor-β, and matrix metallopeptidase-2 that can promote tumor growth. Additionally, microglia-induced increased expression of platelet-derived growth factor receptors in tumor cells can accelerate tumor progression [125, 126]. Studies have also demonstrated that depleting microglia can attenuate malignant glioma growth in mice [125, 127]. Therefore, it is essential to disrupt the communication between glioma cells and microglia or use radiation and/or chemotherapy to prevent the promotion of tumor growth and the creation of new functional states with different abilities to promote tumor growth.

##### Neutrophil

More than 70% of human glioma samples exhibit significant infiltration of neutrophils, which is associated with the grade of the tumor. Neutrophils are capable of permeating the BBB and accessing glioma cells. Furthermore, tumor-associated neutrophils (TANs) have been observed to reside in the vicinity of malignant glioma cells, promoting the recruitment of additional circulating neutrophils. Additionally, the surgical removal of a tumor leads to local brain inflammation, which further facilitates the recruitment of neutrophils. This phenomenon has laid the foundation for the potential application of neutrophil-based DDS in the treatment of brain glioma. Xue et al. [103] creatively reported the use of neutrophils carrying PTX-loaded cationic liposomes (CL) (PTX-CL/NEs) to suppress postoperative glioma recurrence. The highly concentrated inflammatory signals in the brain not only guide the movement of neutrophils into the inflamed brain but also trigger the release of liposomal PTX from the neutrophils, allowing for the delivery of PTX into the remaining invading tumor cells. The results have shown that PTX-CL/NEs present superior inhibitory effects on tumor recurrence in surgically treated glioma mouse models but not in mice with primary gliomas. This indicates that the amplification of inflammatory signals after surgery facilitates the brain tumor targeting and therapeutic efficacy of PTX-CL/NEs. In the following year, Wu et al. [104] clarified the location and behavior of neutrophils after internalizing drug cargoes in the glioma model. In an inflamed mouse glioma model, systemically injected neutrophil carriers can migrate outside the vasculature and move to the inflamed glioma sites along the gradients of molecular guidance signals (chemoattractants or chemokines). Subsequently, the cargoes were unloaded from the neutrophil carriers through neutrophil extracellular trap formulation, but not through exosome secretion in the inflammatory region. Finally, the released cargos were taken up by the glioma cells and performed anticancer efficacy [104]. Thus, it is important for the anticancer efficiency of nanoparticles that the neutrophil carriers accumulate at the inflammatory vascular site and that the drug is accurately unloaded from the neutrophil carriers in the glioma region. In 2021, dual-responsive biohybrid neutrophil microbots were developed. These microbots were equipped with PTX-loaded magnetic nanogels and *Escherichia coli* membrane, which conferred them with magnetic actuated activity. The magnetically actuated intravascular motion and chemotactic behavior along the gradient of inflammatory factors, combined with the inherent chemotaxis of natural neutrophils, greatly enhance the accumulation of PTX in postoperative glioma [105]. In another research, external ultrasound irradiation can be introduced to release PTX from the nanoparticles in the neutrophil carriers on-demand at glioblastoma sites [106]. Specifically, the nanoparticles can generate reactive oxygen species under external ultrasound irradiation, leading to the instability of the liposomal bilayers and the leakage of PTX from the nanoparticles. This property of neutrophil carrier-internalized nanoparticles resulted in the rapid release of PTX from the carriers under external ultrasound irradiation. Although neutrophil-based DDS is an attractive option for treating glioma, there is an ongoing debate about the role of neutrophil carriers in the TME due to the diverse functions of neutrophils, which can have both pro- and anti-tumor effects. The TANs have been categorized as either antitumorigenic (N1) or protumorigenic (N2) TANs (Fig. 5b). Recently, researchers have utilized neutrophil-derived membranes and exosomes as carriers, which not only preserve the distinct functions of neutrophils such as inflammatory chemotaxis and BBB penetration but also mitigate the potential risk of a switch from N1 to N2 TANs during tumor progression [107, 108]. It is worth noting that the neutrophils and their components applied in DDS for targeting glioma were obtained from the peripheral blood or bone marrow of mice. The different phenotypes of neutrophils used in biomimetic DDS may significantly influence their in vivo behavior.

##### NK cell

In addition to macrophages and neutrophils, other types of leukocytes, such as NK cells and DC, also play important roles in the immunosurveillance of glioma (Fig. 5c and d). The peripheral NK cells can identify abnormal cells including tumors, without the need for prior exposure to specific antigen. Moreover, peripheral NK cells can eliminate infected and/or malignant cells through the production of cytokines, perforin, and granzyme, as well as by interacting with apoptotic receptors on target cells using CD95-ligand and tumor necrosis factor-α (TNF-α) [128]. Despite their relatively low levels in TAM, NK cells are considered promising candidates for future therapeutic approaches for gliomas due to their unique characteristics [129]. The infiltration of NK cells into TAM involves interactions between integrin on lymphocytes and ICAM-1 or VCAM-1 on endothelial cells, which result in the disruption of TJs and reorganization of the actin cytoskeleton, leading to the formation of intercellular gaps at the BBB [130, 131]. This characteristic can be exploited by using NK cell membrane-coated nanoparticles. Deng et al. [109] investigated the ability of NK@AIEdots, which are NK cell membrane-coated AIE-active polymeric nanoendoskeletons, to cross BBB in vitro and in vivo. The study demonstrated the presence of LFA-1 and VLA-4 on NK@AIEdots. The efficiency of BBB crossing by NK@AIEdots was approximately 8 times higher than that of naked AIEdots in a BBB model in vitro*.* The inhibiting effect of anti-LFA-1 and anti-VLA-4 antibodies on the BBB crossing efficiency of NK@AIEdots further confirmed the critical role of LFA-1 and VLA-4 proteins in the BBB crossing of NK@AIEdots [109]. Meanwhile, the upregulation of actomyosin stress fiber, which mediates cell motility and contraction, and the downregulation of zonula occludens-1, a TJ-associated protein that maintains endothelial cells morphology and TJ integrity, indicated that NK@AIE dots could serve as TJ modulators to disrupt TJs and reorganize the actin cytoskeleton, thus forming an intercellular “green channel” to facilitate their own crossing of the BBB [109]. Similarly, in nude mice bearing orthotopic glioblastoma U-87 MG, NK@AIEdots showed more pronounced accumulation in the brain and tumor compared to naked NK@AIEdots. The tumor-targeting ability of NK@AIEdots was closely associated with the presence of NKG2D and DNAM-1 on the NK@AIEdots. The recognition and interaction of DNAM-1 and NKG2D with the poliovirus receptor Nectin-2 and major histocompatibility complex class I-related and stress-inducible molecules, which are overexpressed in tumors, resulted in the higher accumulation of NK@AIEdots in U-87 MG glioma cells compared to AIEdots. However, the therapeutic potential of NK cell membranes for treating glioma, particularly due to the expressed CD95-ligand and TNF-α has not been thoroughly investigated, despite the consideration of chimeric antigen receptor NK cell therapy as an effective treatment modality for malignant tumors.

##### DC

The use of DC-based immunotherapy has shown promise as a potential treatment for glioblastoma by stimulating T lymphocyte-mediated anti-cancer immunity [132]. Mature DCs, when stimulated by tumor-associated antigens, can promote the proliferation and activation of CD8^+^ (cytotoxic T cells) and CD4^+^ T lymphocytes (helper T cells), which are involved with tumor apoptosis [132]. Although DC vaccines have shown promising results in clinical trials, the effectiveness of anti-glioblastoma immunotherapy is hindered by glioblastoma-induced immunosuppression and signaling of tumor-associated antigens. Glioblastoma is characterized by an immunosuppressive TME, with cancerous glioblastoma cells initiating this immunosuppression. Nano-DOX has been found to effectively stimulate damage-associated molecular patterns derived from glioblastoma cells, leading to a shift in the immunosuppressive phenotype of glioblastoma-associated macrophage to an immunostimulatory phenotype [92]. On this basis, Li et al. [110] utilized DC-mediated delivery of Nano-DOX (Nano-DOX-DC) to explore the potential of overcoming the glioblastoma immunosuppressive microenvironment and enhancing the DC-driven anti-glioblastoma immune response. Their findings indicated that the intravenously injected Nano-DOX-DC could migrate to the tumors of mice with orthotopic glioblastoma xenografts. In the presence of Nano-DOX-induced glioblastoma cell damage, the infiltrated DC carriers were effectively activated, resulting in an enhanced mouse lymphocyte-mediated immune response [110]. It is worth noting that DC membranes have been found to retain tumor-associated antigens and T-cell stimulating factors, offering the potential for a cytomembrane vaccine [111]. Additionally, tumor cell membranes contain abundant levels of tumor antigens, including tumor-associated and tumor-specific antigens, which can help decrease immune evasion, break immunological tolerance, and target tumor cells [133]. Inspired by these natural features, Hao et al. [112] developed a hybrid membrane‑coated DTX nanosuspension based on DC membranes and C6 cell membranes (DNS-[C6&DC]m) for multi‑modal anti‑glioma therapy. The delivery of tumor antigens derived from the C6 cell membrane efficiently elicited the antitumor immune responses, leading to a significant increase in the expression of CD8 and CD4 at tumor sites and spleen following in vivo injection of DNS-[C6&DC]m [112]. Furthermore, glioma growth was obviously suppressed by DNS-[C6&DC]m in the C6 glioma-bearing mice, indicating that the combination of drug delivery and antigen delivery resulted in a better chemo-immunotherapeutic effect in gliomas [112].

#### Ischemic stroke

**Fig. 6:**
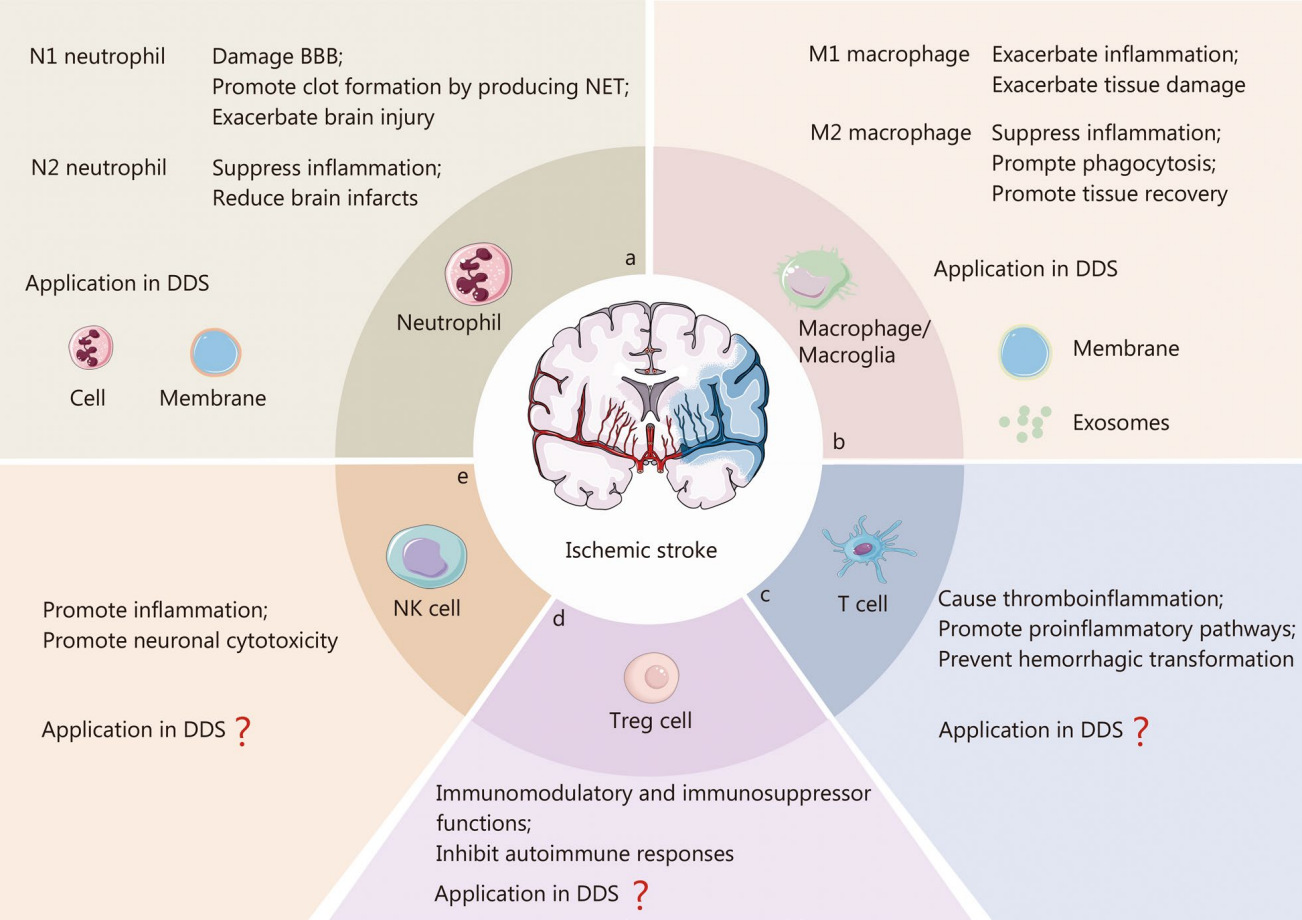
The role of infiltrating immune cells in ischemic stroke and their application or non-application in immune cell-based DDS. This includes neutrophil (**a**), macrophage/microglia (**b**), T cell (**c**), Treg cell (**d**), and NK cell (**e**). BBB blood–brain barrier, DDS drug delivery systems, NETs neutrophil extracellular traps, NK natural killer. “?” represents no current application in DDS

Ischemic stroke is characterized by necrosis caused by local blood supply obstruction in the brain, with high morbidity, disability, and mortality rates [134]. Current clinical treatments for ischemic stroke primarily involve endovascular thrombectomy and intravenous thrombolytic drug administration [135]. However, the narrow treatment windows and low treatment rates for stroke patients [136] underscore the importance of identifying effective neuroprotective therapy for ischemic stroke. The presence of BBB poses a significant challenge to the delivery of neuroprotective drugs. Recent studies have shown the crucial role of immune cell-mediated immune responses in the pathogenesis of ischemic stroke, including acute intravascular events triggered by blood supply disruption, the inflammatory cascade leading to brain damage, and the subsequent tissue repair phase [137–139]. Following ischemia, the adhesion molecule P-selectin is rapidly deployed to the surface membrane of platelet and endothelial cells, while pro-inflammatory cytokines are promptly generated and released, triggered by the upregulated nucleotides (ATP, UTP) from injured cells like neurons [138]. Concurrently, perivascular macrophages and mast cells become activated, leading to the release of proteases and pro-inflammatory cytokines [138]. The increased proteases can facilitate the extravasation of proteins and cells via the paracellular route by downregulating TJ proteins between endothelial cells [139]. Furthermore, the upregulated pro-inflammatory factors can stimulate the release of various chemokines (CCL2, CCL20, and CXCL2) to attract peripheral immune cells to the ischemic region and increase the endothelial expression of ICAM-1 and VCAM-1, thereby facilitating the infiltration of peripheral immune cells into the damaged brain parenchyma. The recruited peripheral immune cells include neutrophil, macrophage/microglia, lymphocyte, and DC (Fig. 6).

##### Neutrophil

Neutrophils are the initial immune cells to enter the damaged area of the brain during acute ischemic stroke [140, 141]. Following the onset of cerebral ischemia, neutrophils gather in the cerebral microvessels and venules within minutes and gradually move to the ischemic brain tissue, guided by increased secretion of chemokines (CXCL1, CXCL2) by astrocytes [142]. The migration of neutrophils peaks after 24 h and persists for over 32 days [143]. This migration behavior has prompted research into utilizing neutrophils for DDS. Additionally, stroke patients exhibit a significant increase in circulating neutrophils due to a systemic immune response. The abundance of neutrophils presents an opportunity for potential drug delivery to the ischemic region to alleviate ischemic injuries. Hou et al. [144] found that cRGD peptide-modified liposomes have a high affinity for neutrophils, as they recognize integrin avb1, which is abundantly expressed on the surface of neutrophils. This recognition efficiently triggers the uptake of cRGD-modified liposomes by neutrophils [144]. Neutrophils effectively deliver cRGD-liposome to the ischemic region owing to the chemotactic nature in a middle cerebral artery occlusion (MCAO) mice model. The delivery efficiency of the cargo is closely linked to the migration efficiency of neutrophils, which depends on secondary inflammatory signals following the onset of ischemia. Similar to the infiltrated neutrophils in the ischemic region, cRGD-liposome significantly accumulated in the ischemic region as early as 3 h and continued to increase up to 24 h after the ischemic insult [144]. The cargo can be transferred from the neutrophils to neuronal cells through transient intercellular connections and the secretion and fusion of exosomes by neutrophils and neuronal cells [145]. The migration process of neutrophils was dominated by integrin β2, macrophage-1 antigen, and LFA-1 on the neutrophil membrane. Efficient delivery of mesoporous Prussian blue nanozyme and resolvin D2, loaded in neutrophil membrane and neutrophil membrane-derived nanovesicles, respectively, further supports the use of neutrophils for delivery into ischemic regions [146, 147]. Interestingly, reduced recruitment of neutrophils was observed in the neutrophil-like cell-membrane-coated mesoporous Prussian blue nanozyme (MPBzyme@NCM) treated tMCAO/stroke mice at day 5 after stroke, as indicated by Western blotting analyses of myeloperoxidase, a marker for neutrophils. This phenomenon may result from the interaction of neutrophil membrane-coated nanoparticles with adhesion molecules on inflamed brain microvascular endothelial cells [146]. In addition, MPBzyme@NCM-treated tMCAO/R model mice exhibited an increased M2 phenotype macrophage/microglia and decreased apoptosis of neurons [146]. However, it is unclear whether these favorable stroke recovery phenomena are associated with neutrophil membranes. Various reports have suggested that the infiltration of neutrophils is linked to BBB breakdown, edema, and brain infarcts [148, 149]. Neutrophil depletion contributes to brain tissue repair after stroke [150, 151].

##### Macrophage/microglia

It has been documented that the presence of macrophage/microglia (including resident microglia and infiltrated macrophages) in the brain reached its peak approximately 4 days after cerebral ischemia in rats or mice. Resident microglia activation predates and predominates over blood-derived macrophages. Following an ischemic stroke, the release of damage-associated molecular patterns can trigger local immune responses, leading to the activation of glial cells within minutes. The activated microglia primarily exhibit M1-type characteristics, producing and releasing significant amounts of pro-inflammatory factors (IL-1α, IL-1β, IL-6, TNF-α, IFN-γ) [152]. After 1 days, the activated microglia migrated to the infarct core via the annexin-1/casein kinase II pathway [153]. Blood-derived macrophages were recruited into the ischemic brain tissue within 3–4 h after the onset of ischemia. guided by CCL2. The phenotype of infiltrating macrophage/monocytes and activated microglia undergoes dynamic polarized changes [154]. Specifically M1-like cells gradually increased within 14 days after the onset of stroke, while M2-like cells tended to increase in the first 1–2 days and then gradually decreased [152, 154, 155]. Among these, M2-like macrophages promote tissue recovery,d axonal outgrowth, and angiogenesis after ischemic stroke by secreting protective remodeling factors (VEGF, BDNF, progranulin, and transforming growth factor factor-β), anti-inflammatory cytokines, and proteinases [156]. A recent study found that spleen-targeted glabridin-loaded nanoparticles (NPGla-5k) could effectively regulate the polarization of macrophages/monocytes in the spleen into M2-macrophages, accompanied by the infiltration of peripheral macrophages into the ischemic penumbra after tail-vein injection. NPGla-5k treatment can effectively reduce inflammatory damage, protect damaged neurons, and improve nervous system function in MCAO/R mice [157]. Similarly, macrophage-derived exosomes exhibited superior migration capability to cross the BBB and accumulate in the ischemic brain with the loaded drugs [158, 159]. Meanwhile, the microglia and neuronal cells can internalize the drug-loaded migrated exosomes [158]. Surprisingly, lipopolysaccharide (LPS)-induced macrophage exosomes exert therapeutic effects on ischemic stroke by promoting microglia polarization from M1 to M2 [160]. Macrophage-derived membrane-based biomimetic nanoparticles have also made some progress in the treatment of ischemic stroke. These biomimetic nanoparticles were endowed with the natural targeting and migration ability of macrophages to the ischemic brain due to the preservation of most cell membrane surficial proteins, including CD11b, CD44, integrin α4, and integrin β1, as evidenced by the distribution of these biomimetic nanoparticles in the ischemic brain markedly beyond that of the naked nanoparticles [161–164]. Interestingly, microglia-derived membrane-based biomimetic nanoparticles also demonstrated the ability to cross the BBB, though there were no reports about the migration of microglia from peripheral to CNS [165]. Moreover, the M2 microglia membranes can serve as bioinspired therapeutic agents to repolarize M1 microglia into the M2 phenotype, which may result from the presence of anti-inflammatory proteins on the membrane, such as CD206 [165].

##### Lymphocyte

Lymphocytes, including T cells, Treg cells, and NK cells, among others, were observed to migrate to the ischemic brain following an ischemic event [166]. While lymphocytes represent a small proportion of the infiltrating immune cells. T cells were found to migrate from subpial and cortical vessels as well as choroid plexus to the infarcted hemisphere on day 1 after ischemia, with this process persisting for an extended period [167]. Peripheral T cells were shown to have a significant impact on preventing hemorrhagic transformation in severe ischemic stroke by interacting with platelets [168]. Infiltrating T cells have been related to promoting the proinflammatory pathway, while another infiltrating lymphocyte, NK cells, have been found to exhibit pathogenic actions in ischemic stroke, including promoting inflammation and neuronal cytotoxicity [169]. In the ischemic cerebral hemisphere, the number of NK cells rapidly increased and peaked at 3 h post-ischemia, and then decreased [169]. Conversely, regulatory lymphocytes, such as Tregs and regulatory B cells (Bregs), have been shown to contribute beneficially to recovery following ischemic stroke [170]. Treg cells were observed to accumulate in the ischemic lesion at 15 days and persist at 30 days post-stroke [171]. Attenuation of Treg cells function resulted in aggravated tissue loss and impaired neurological function in MCAO mice. In contrast, enhancing the number or functions of Treg cells was found to be conducive to recovery [172]. These results support the potential of Treg cell therapy. Up to now, there have been no reported studies on Treg cell-based DDS in the treatment of ischemic stroke. The application of immune cell-based DDS in ischemic stroke is summarized in Table 3 [144–147, 157–165].

**Table 3:** Application of immune cell-based DDS in ischemic stroke

Immune cells	Components	Loaded drugs/nanoparticles	Methods	Model	Functions	References
Neutrophil	Cell	cRGD-modified liposome (Edaravone)	Trojan horse	MCAO model	–	[144]
	Cell	Neutrophil-targeted polymeric nanoparticles	Trojan horse	MCAO model	–	[145]
	Membrane	Mesoporous Prussian blue nanozyme	Hypotonic lysis and homogenizer Co-extrusion	MCAO model	Reduce the recruited neutrophils Promote microglia polarization from M1 to M2 Decrease apoptosis of neurons Upregulate neurogenesis	[146]
	Nanovesicles	Resolvin D2	Centrifugal separation; Sonication and incubation	MCAO model	Suppression of inflammation	[147]
Macrophage	Cell	Spleen-targeted glabridin-loaded nanoparticles	Trojan horse	MCAO model	Enhance the polarization of Mo/Mϕ into M2-macrophages in the spleen Inhibit the inflammatory cascade in the penumbra	[157]
	Exosomes	Edaravone	Incubation and centrifugal separation (pre-loading)	MCAO model	Reducing the damage and death of neuronal cells Promote the polarization of microglia from M1 to M2	[158]
	Exosomes	CUR	Incubation and centrifugal separation (pre-loading)	MCAO model	Alleviate BBB damage Suppress mitochondria-mediated neuronal apoptosis	[159]
	Exosomes	LPS-induced macrophage exosomes	Ultrafiltration	MCAO model	Promote the conversion of microglia from M1 to M2 phenotypes Reduced M1 microglia-induced neuronal toxicity	[160]
	Membrane	Fingolimod-loaded MnO_2_ nanoparticles	Hypotonic lysis, freezing and thawing, and homogenizer Co-extrusion	MCAO model	Reduce oxidative stress Modulate inflammatory microenvironment Reinforce the survival of damaged neuron	[161]
	Membrane	Tetramethylpyrazine-loaded ROS-responsive nanoparticles	Lysis buffers and homogenizers Co-extrusion	MCAO mode	Eliminate ROS Promote the regeneration of neural cells Suppression of inflammation	[162]
	Membrane	CUR-loaded ROS-responsive nanoparticles	Lysis buffers and homogenizers Co-extrusion	MCAO model	Eliminate ROS Promote the regeneration of neural cells Suppression of inflammation	[163]
	Membrane	BA loaded liposome	Sonication and centrifugal; Co-extrusion	MCAO model	Improve the circulation of BA in blood Improve neuroprotective effect	[164]
Microglia	Membrane	Catalase-loaded acid-responsive nanoparticle	Lysis buffers and co-extrusion	MCAO model	Eliminate excessive Fe^2+^ in-situ Eliminate ROS Promote microglia polarization from M1 to M2	[165]

*BA* baicalin, *BBB* blood–brain barrier, *cRGD* cyclo (Arg-Gly-Asp-d-Tyr-Lys), *CUR* curcumin, *LPS* lipopolysaccharide, *Mo/Mφ* monocyte/macrophage, *MCAO* middle cerebral artery occlusion, *ROS* reactive oxygen species. “–” represent not mentioned

#### Neurodegenerative diseases

**Fig. 7:**
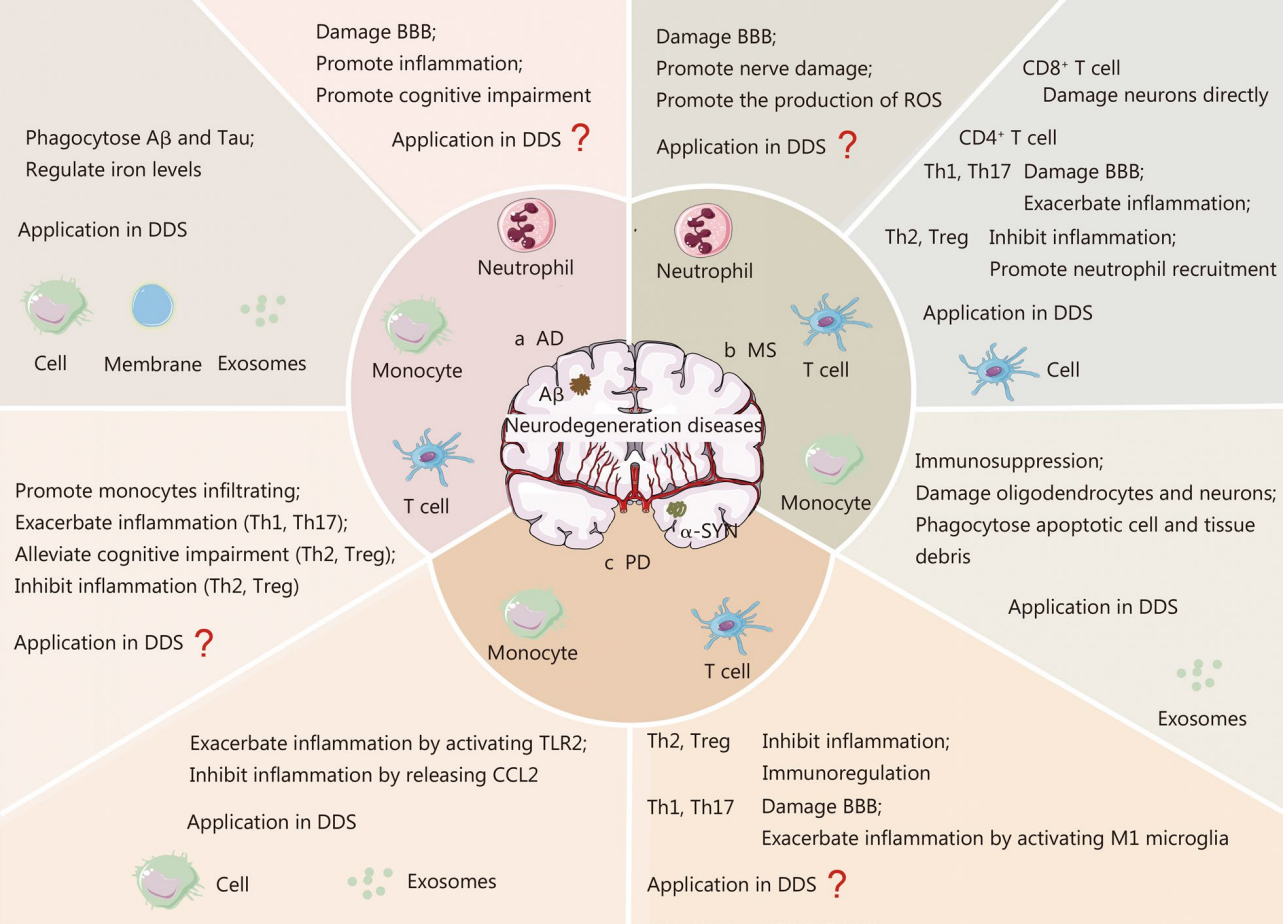
The role of infiltrating immune cells in neurodegenerative diseases and their application or non-application in immune cell-based DDS, including AD (**a**), MS (**b**), and PD (**c**). AD Alzheimer’s disease, Aβ amyloid-β, BBB blood–brain barrier, CCL2 c–c motif chemokine ligand 2, DDS drug delivery systems, MS multiple sclerosis, PD Parkinson’s disease, ROS reactive oxygen species, TLR2 toll-like receptor 2. “?” represents no current application in DDS

AD and PD are the most prevalent neurodegenerative diseases, with AD being more common among the elderly, affecting around 10% of individuals over 65. AD is characterized by accumulated amyloid-β (Aβ) plaques, neurofibrillary tangles, and neuronal loss, leading to progressive cognitive deterioration. Accumulating evidence indicates that the amyloid cascade hypothesis and tau protein, although well-established, may not be the sole causative factors in AD. The presence of inflammatory markers in AD patients suggested an association between immune cells and pathological processes underlying AD [173–175]. Microglia, as innate immune cells, play a complex role in AD pathogenesis through various activation pathways [176]. They display diverse phenotypes and engage in multifaceted interactions with Aβ and tau species, as well as neuronal circuits [176]. Microglia recruited to the sites of Aβ plaque deposition can phagocytose Aβ, thereby facilitating its elimination [177]. Moreover, soluble hyperphosphorylated tau protein can induce degeneration of microglia, impairing their immunosurveillance function and promoting the formation of neurofibrillary tangles [178]. Typically, these neurofibrillary tangles are internalized by microglia [179]. Although AD brains generally have fewer peripheral immune cell infiltrations compared to glioma or other neuroinflammatory diseases like MS [180], evidence indicates that under Aβ stimulation, immune cells such as neutrophils, monocytes, and T cells can infiltrate the brain (Fig. 7) [181].

##### AD

In the brains of AD transgenic mice, there is a notable accumulation of monocytes specifically around Aβ plaques. Unlike in other neurodegenerative diseases, the monocytes that infiltrate the AD brain have a positive impact on the disease’s progression by limiting Aβ plaques [182]. This unique ability of monocytes to enter the brain and aid in Aβ elimination has led to the exploration of monocyte or its differentiated cells (including macrophages and microglia)-based delivery systems for proteins, miRNA, and drugs. Böttger et al. [183] utilized Bioporter™ to facilitate the recombination of NGF-loaded primary rat monocytes. The results revealed that over 30% of NGF-loaded monocytes were able to adhere to the monolayer of rat brain capillary endothelial cells (BCEC) and efficiently traverse a simplified artificial BCEC monolayer. Additionally, the loaded NGF can be released by migrating monocytes into the basolateral medium, thereby providing neuroprotection for cholinergic neurons against degeneration [183]. Furthermore, the migration ability of monocytes loaded with proteins, miRNA, and drugs, was further demonstrated. Moreover, monocyte-derived membranes, exosomes, and EVs have been found to serve as effective transport vehicles across the BBB in AD model mice [184–186]. The mechanism of exosome-loaded drugs’ migration was investigated, revealing that the specific interaction between monocyte-derived exosomes’ LFA-1 and endothelial ICAM-1 mediates their migration across the BBB. Although there was no obvious evidence that the monocyte-derived exosomes specifically accumulate around specific brain cells, they have been shown to fuse with astrocytes and deliver drugs to specific brain regions [186].

To improve the delivery efficiency of exosomes- or membrane-based DDS for targeting neurons, microglia, or organelles such as lysosome and mitochondria, various chemicals, proteins, and antibodies like rabies virus glycoprotein (RVG29), mannose, or triphenylphosphine cation (TPP) can be modified on the surface of the exosomes. Mitochondrial dysfunction is a contributing factor to the production and abnormal aggregation of Aβ, thus leading to neuron apoptosis. Han et al. [187] used RVG29 and TPP-modified macrophage membrane-coated solid lipid nanoparticles (SLNs) to deliver Genistein (GS) (RVG/TPP-MASLNs-GS), a natural flavonoid that effectively inhibits neuronal apoptosis induced by Aβ in vitro, to abnormal mitochondria in the neuron. The attachment of RVG29 and TPP on the macrophage membrane did not affect the macrophage membrane’s ability to evade the reticuloendothelial system, as evidenced by similar pharmacokinetic curves and parameters between MASLNs-GS and RVG/TPP-MASLNs-GS groups [187]. Additionally, RVG29 modification reinforced the macrophage membrane’s innate ability to cross the BBB, a crucial requirement for RVG/TPP-MASLNs-GS to target neuronal mitochondria [187]. In this research, MASLNs presented poor BBB permeability in an in vitro BBB model and in vivo imaging, likely due to the absence of chemokines. Conversely, the significant signal was observed in the lower neuron of the BBB model in the RVG-MASLNs and RVG/TPP-MASLNs groups, consistent with in vivo imaging results. Notably, a large number of RVG/TPP-MASLNs were internalized by neurons rather than astrocytes in the bEnd.3/HT22 or bEnd.3/astrocytes co-culture BBB models, as RVG29 exhibits higher selectivity for neurons [188, 189]. Furthermore, RVG/TPP-MASLNs effectively inhibit mitochondrial reactive oxygen species in Aβ-treated HT22 neuronal cells, facilitated by TPP-mediated mitochondria targeting [190].

Interestingly, exosomes can attach to Aβ because of the high presence of GM1 ganglioside in their membranes. This is known to cause conformational changes in Aβ, leading to the formation of non-toxic amyloid fibrils and promoting the absorption of Aβ [191–194]. Additionally, microglia with impaired lysosomal function are associated with abnormal endocytosis and insufficient clearance of Aβ through lysosome-mediated degradation. Building on this, Hao et al. [185] developed mannose-conjugated macrophage-derived exosomes (MExo) to promote the targeted delivery of gemfibrozil (Gem) and restore the lysosomal activity of microglia in clearing Aβ aggregation. MExo-Gem was found to reduce the formation of amyloid fibrils by binding to Aβ and delivering Aβ precisely to microglia via the interaction between the mannose modified on exosomes and mannose receptors expressed in microglia [195]. This was demonstrated by a decrease in Thioflavin T fluorescence intensity and an increase in the concentration of Aβ in microglia in the MExo-treated group [185]. The accumulated Gem effectively restored lysosomal activity by promoting the nuclear translocation of transcription factor EB and activating peroxisome proliferator-activated receptor α. The activated lysosomes in microglia contributed to accelerating lysosome-mediated clearance of the accumulated Aβ in microglia, as demonstrated in vivo and in vitro. Finally, MExo-Gem significantly reduced neuronal injury and improved learning and memory ability, as evidenced by the highest target quadrant occupancy and crossing numbers in Aβ-induced AD mice [185]. In contrast to monocyte-derived components (exosomes, EVs, or membranes), an excess of monocytes may have uncontrolled effects in the brain as carriers of the BBB, and it is unclear whether an increase in macrophages/microglia in the brain leads to beneficial or harmful effects in the context of nerve injury.

In contrast to macrophages, the infiltrating neutrophils and T lymphocytes play an important role in the development of AD [196, 197]. Activated circulating neutrophils cause damage to BBB and neurotoxicity in AD by producing inflammatory mediators such as myeloperoxidase and reactive oxygen species, and by releasing neutrophil extracellular traps (NETs) [198, 199]. In AD models, reducing the number of neutrophils or inhibiting their movement through blocking LFA-1 or integrins α4 can decrease AD-like neuropathology and improve cognitive function [200–202]. Infiltrating T cells were classified as CD4^+^ T cells and CD8^+^ T cells, with CD8^+^ T cells infiltrating the CNS and causing harmful effects [196]. The effects of infiltrating CD4^+^ T cells varied depending on their phenotypes. Specifically, Th1 and Th17 cell types may have negative effects on nearby neurons or glial cells by producing pro-inflammatory cytokines, while Th2 or Treg cells may suppress neuroinflammation in AD by secreting anti-inflammatory cytokines or through cell–cell contact [194]. Additionally, the increased expression of CCR on T cells and their corresponding ligands in the AD brain further facilitates the recruitment of peripheral immune cells into the CNS [196]. The application of immune cell-based DDS in AD is outlined in Table 4 [59, 183–187].

**Table 4:** Application of immune cells based on DDS in AD

Immune cells	Components	Loaded drugs/nanoparticles	Methods	Model	Functions	References
Monocyte	Cell	Nerve growth factor	Trojan horse	Brain capillary endothelial cell monolayer	Migrate into brain slices	[183]
Macrophage	Exosomes	Curcumin	Incubation and ultracentrifugal separation (pre-loading)	Stereotaxic-induced AD model	Target BBB and hippocampal nerve cells Inhibit hyperphosphorylation of Tau protein Ameliorate cognitive function	[184]
	Exosomes	Silibinin	Incubation and differential ultracentrifugal separation (pre-loading)	Aβ1-42-induced AD model	Inhibition of Aβ aggregation Activate astrocytes Alleviate cognitive impairment	[186]
	RVG/TPP-modified membrane	MASLNs	Hypotonic lysis and centrifugal separation; Co-extrusion	APP/PS1 mice	Target BBB and neuronal mitochondria	[187]
Microglia	Mannose-modified EVs	Gemfibrozil	Incubation and gradient centrifugation separation (pre-loading)	Aβ1-42-induced AD model	Target BBB and microglia Facilitate Aβ clearance via enhancing activity of microglial lysosome; Promoting cognitive recovery	[185]
DC cell	Lamp2b overexpressing and RVG-fused exosomes	siRNA	Differential centrifugal separation and electroporation (post-loading)	BACE1 knockdown model mice	Target BBB, neurons, microglia, and oligodendrocytes Decrease amyloid plaques Induce immune responses	[59]

*AD* Alzheimer’s disease, *Aβ* amyloid-β, *APP/PS1* APPswe/PSEN1dE9, *BACE1* β-secretase 1, *BBB* blood–brain barrier, *EVs* extracellular vesicles, *MASLNs* macrophage membrane-coated solid lipid nanoparticles, *RVG* rabies virus glycoprotein, *TPP* triphenylphosphine

##### PD

PD is characterized by the significant loss of dopaminergic neurons in the substantia nigra and the aggregation of α-synuclein in Lewy bodies [203]. Additionally, there is evidence of inflammation and activation of microglia, as represented in postmortem tissue studies of PD patients and an increase in cytokines in the brain and CSF [204]. The activated microglia and astroglia released pro-inflammatory cytokines, leading to endothelium-derived inflammatory responses and the expression of adhesion molecules [203], which in turn recruit circulating blood cells into the brain microvasculature. Furthermore, there is an innate immune response in the periphery triggered by the antigenicity of peripheral α-synuclein. Post-mortem brain samples of PD patients have shown the presence of infiltrating immune cells, including T cells, NK cells, and monocytes/macrophages, in the substantia nigra [205, 206]. The interaction between immune cells in the periphery and the brain not only influences the overall immune response in PD but also provides a potential avenue for delivering proteins, mRNA, and drugs into the brain [207–219].

Batrakova et al. [40] and other researchers utilized a bone-marrow-derived macrophage system to deliver catalase to brain regions affected by PD [207–209]. They encapsulated catalase/PEI-PEG complexes into the macrophages to protect catalase from degradation and ensure its sustained release [40, 207]. The loaded macrophages were observed to move along microvessels, adhere to endothelial walls, and cross the BBB into the parenchyma during brain inflammation, [208]. Compared to free nanozyme, more nanozyme was transferred from the loaded macrophage to various target cells, including endothelial cells, neurons, and astrocytes, leading to decreased reactive oxygen species, reduced neuroinflammation, and protection against degeneration. The enhanced transfer of nanozyme from macrophages to target cells involved the fusion of cell membranes, the formation of macrophage bridging conduits, and the release of exosomes containing nanozyme [209]. In this study, macrophages loaded with catalase were utilized as depots, while subsequent investigations subtly advanced the development of macrophage carriers as production facilities. Genetically modified macrophages were constructed by transfection of pDNA encoding catalase. The transfected gene enables these macrophages to effectively reach the brain and prolong the secretion of catalase in the brain of PD mice [210]. Interestingly, the exosomes from catalase-transfected macrophages efficiently transferred their contents (DNA, mRNA, transcription factors molecules, and the encoded protein) to neighboring neurons [210] leading to sustained catalase expression and contributing to anti-inflammatory and neuroprotective effects in murine models of neuroinflammation and PD.

In addition to the efficient delivery of catalase and catalase pDNA into the brain of PD model mice via macrophages, these macrophages when transfected with glial cell line-derived neurotrophic factor (GDNF) were recruited to substantia nigra during neurodegeneration. This recruitment significantly improved neurodegeneration and neuroinflammation in 6-hydroxidophamine (6-OHDA)-intoxicated mice and transgenic Parkin Q311X(A) mice [211–216]. The accumulated GDNF-transfected macrophages were able to differentiate into the regenerative M2 phenotype. Furthermore, the GDNF formed by these macrophages could transfer to target neurons, facilitated by the targeted ability of macrophage-derived exosomes containing GDNF. Similarly, bone marrow-derived microglia transfected with neurturin could also cross the BBB and were recruited in large numbers to sites of neurodegeneration, where they become activated microglia capable of secreting trophic factors [217]. It has been noted that the targeted ability of exosomes or EVs is related to the cell source. A previous study has shown that the brain accumulation levels of mEVs in a transgenic mouse model of PD are significantly higher than nEVs and aEVs [80]. Research using mEVs as the carriers of catalase for PD treatment has further demonstrated the targeted ability of these mEVs to reach inflamed brain tissues [218]. The application of immune cell-based DDS in PD is summarized in Table 5 [40, 207–219].

**Table 5:** Application of immune cells DDS applied in PD

Immune cells	Components	Loaded drugs/nanoparticles	Methods	Model	Functions	References
Macrophage	Cell	Catalase/PEI-PEG complexes	Trojan horse	MPTP-intoxicated mice BBB model in vitro	Target affected brain subregions in models of PD and transport the loaded drug from macrophages to endothelial, neuronal, and glial target cells Reduce oxidative stress Reductions microglial activation and astrocytosis Increase survival of dopaminergic neurons and nigrostriatal NAA levels	[40, 207–209]
	Cell	Catalase pDNA	Gene transfection	6-OHDA or LPS-induced brain inflammation model	Target BBB and transfer the loaded drug to neural cells Reduce inflammation Increase neuroprotection Improve motor functions	[210]
	Cell	GDNF	Gene transfection	MPTP-intoxicated mice MitoPark mouse 6-OHDA-intoxicated mice Transgenic Parkin Q311X(A) mice	Ameliorate neurodegeneration and neuroinflammation Stimulate axon regeneration Diminish alpha-synuclein aggregation Increase survival of dopaminergic neurons Improve motor and non-motor dysfunction	[211–215]
Bone marrow-derived-microglia	Cell	Neurturin	Lentiviral transduction	MPTP-intoxicated mice	Protect dopaminergic neurons Ameliorate neurodegeneration and dopaminergic injury Improve motor dysfunction	[217]
Macrophage	Exosomes	GDNF	Gene transfection and differential centrifugation	Transgenic Parkin Q311(X)A mice	Improve mobility Increase neuronal survival Decrease neuroinflammation	[216]
Macrophage	Exosomes	Catalase	Differential centrifugation and filtration; incubation at RT with or without saponin Freeze/thaw cycles technique Sonication Extrusion	6-OHDA-intoxicated mice	Target neuronal cells Protect substantia nigra neurons against oxidative stress	[218]
Macrophage	Nanovesicles	Curcumin	Recycled exclusion and sonication	MPP^+^-induced neuronal degeneration model	Target BBB and neurons Ameliorate neurodegeneration	[219]

*6-OHDA* 6-hydroxidophamine, *BBB* blood–brain barrier, *GDNF* glial cell line-derived neurotrophic factor, *LPS* lipopolysaccharides, *MPP*^+^ 1-methyl-4-phenylpyridinium, *MPTP* 1-methyl-4-phenyl-1,2,3,6-tetrahydropyridine, *NAA* N-Acetyl-L-aspartic acid, *PD* Parkinson’s disease, *pDNA* plasmid DNA, *PEI-PEG* polyethyleneimine-poly (ethylene glycol), *RT* room temperature

##### MS

MS is a chronic autoimmune, inflammatory, and neurodegenerative disease that affects more than 2.5 million people worldwide. The disease is considered to be triggered by the activation of CNS-reactive T cells in the periphery, which are then arrested by endothelial cells in the CNS and subsequently migrate across the BBB into the CNS [197, 220]. This process involves the expression of chemokines, integrin, and selectin by inflammatory endothelial cells, leading to the recruitment and migration of immune cells [220]. The resulting immune dysregulation in the CNS is the primary cause of MS, leading to demyelination, axonal damage, and neurodegeneration [221]. Similar to AD, the pathogenesis of MS involves the activation of microglia and astrocytes, resulting in the release of pro-inflammatory cytokines and chemokines, which in turn recruit more immune cells from the periphery to the CNS [220]. The main therapeutic objective for MS is to suppress the immune response in the CNS. There are three treatment strategies approved by the Food and Drug Administration (FDA) for MS patients: regulating the immune state to induce tolerance, blocking T cell trafficking to the CNS, and inhibiting T cell division and proliferation [222]. However, these non-specific immunosuppressive treatments may pose serious risks in the medium to long term. As a result, numerous nanocarriers have been designed to target peripheral immune cells, such as macrophages, DC, and B cells, to deplete monocytes, induce specific antigen tolerance, or deliver corticosteroids. Additionally, immune cells have been utilized as “Trojan horse”-mediated DDS to efficiently migrate into the CNS parenchyma, overcoming systemic treatment dispersion, and reducing the number of B cells in spinal cord infiltrates [223]. One specific example is the development of a T cell-mediated DDS, where iron-oxide nanoparticles (NBR) conjugated with the monoclonal antibody anti-CD20 (NBR-anti-CD20) were loaded in MOG35-55 antigen-specific T cells. Anti-CD20 is the only FDA-approved disease-modifying therapy for primary progressive MS and depletes B cells [224]. This approach has shown promise in depleting B cells and preserving neurons and the axonal state in experimental autoimmune encephalomyelitis mice. [223].

Immune cell-based DDS has also been applied in epilepsy and depression, both of which involve neuroinflammation [225, 226]. The application of immune cell-based DDS in MS, depression, and epilepsy is further detailed in Table 6 [223, 225, 226].

**Table 6:** Application of immune cell-based DDS in MS, depression, and epilepsy

Immune cells	Components	Loaded drugs	Methods	Model	Functions	References
T cell	Cell	NBR functioned with an anti-CD20 monoclonal antibody	Trojan horse	Experimental autoimmune encephalomyelitis	Deplete B cells in the spleen and the brain Ameliorates the disease course and pathology	[223]
Monocyte	Cell	cRGD-modified liposome	Trojan horse	IL-1β-induced brain inflammatory model mice The CMS-induced depression model mice	Target basolateral amygdala regions Antidepressant	[225]
Monocyte	Cell	Polylactic acid-based magnetite-impregnated MNPs	Trojan horse	Spontaneous recurrent seizures model rats	Target the hippocampal CA1 and dentate gyrus	[226]

*CA1* cornu ammonis 1, *CMS* chronic mild stress, *cRGD* cyclic RGD, *DDS* drug delivery system, *IL-1β* interleukin-1β, *MNPs* monocyte nanoparticles, *MS* multiple sclerosis, *NBR* magnetite (Fe_3_O_4_) nanoparticles

## Conclusion and future perspectives

The interaction between immune cells and the CNS during disease provides a potential chance for delivering drugs to the brain. Substantial data have demonstrated the potential of immune cell-based DDS, which involves using immune cells to transport nanoparticles into the brain. These strategies include immune cells carrying nanoparticles (hitchhiking nanoparticles, immune trojan horses, immune cells with backpacks), nanoparticles coated with the immune cell membrane, and exosomes derived from immune cells loaded with nanoparticles. These methods take advantage of the natural migratory ability of immune cells across the BBB due to receptors on their membranes. However, it's important to consider the role of immune cells in CNS diseases when using these strategies. The effectiveness and properties of these DDS depend on the specific immune cells chosen. Here are the factors to consider in the design process of immune cell-based DDS. (1) The number and timing of immune cells infiltrating are crucial factors determining the efficiency of immune cell-based DDS delivery. (2) The migrated mechanism of immune cells is mainly mediated by adhesion molecules on the endothelial cells, such as VCAM, ICAM, and P-selectin, as well as their integrin ligands on immune cells like LFA, Mac, and selection. Additionally, other upregulated adhesion molecules on endothelial cells can also serve as ligands for immune cell-based nanoparticles to improve CNS barrier penetration. Based on this premise, targeting multiple adhesion molecules through genetic engineering modification can enhance the penetration of immune cell-based DDS into the BBB. Interestingly, chemokine receptors can strengthen the interactions between integrin and cell adhesion molecules by mediating integrin clustering and conformational changes during the migration of immune cells. When designing immune cell-based nanoparticles, exogenous modification or construction with upregulation of chemokine receptors can be employed. (3) The function of immune cells is vital in the pathogenesis of CNS diseases. It is crucial to understand the role of cell-mediated immunity to consider leveraging the immunotherapeutic potential of immune cells beyond just their migratory abilities. (4) Safety. The infiltration of exogenous immune cell-based nanoparticles into the CNS, especially immune cell carriers, can potentially preserve the functional immune response of specific immune cells. Simultaneously, targeted integration of immune cell-based nanoparticles with classical cell adhesion molecules may effectively inhibit CNS infiltration by immune cells. These actions have the potential to interfere with the host immune response or result in serious side effects.

This review highlights the potential of using immune cell-based nanoparticles for treating CNS diseases. While the findings are promising, they are primarily based on laboratory studies. The translation of these nanoparticles into clinical use faces significant challenges. (1) Standard procedures are crucial for quality controls, including cell culture, cell membrane and exosome extraction, and nanoparticles package. Precise and reproducible procedures are necessary to maintain the functions of these nanoparticles, such as immune evasion, targeting, and immunomodulatory effects. (2) Strict control over storage conditions (solvent type, particle concentration, freeze-drying method, temperature, and reconstituted condition) is essential to preserve protein activity. (3) Ensuring the immune safety of these nanoparticles, particularly immune cell carriers are vital, and their effects on different immune cell subsets should be thoroughly investigated to minimize potential side effects. Overall, the migration of immune cells to CNS presents an opportunity for drug delivery using immune cell-based nanoparticles and other strategies.

## Data Availability

Not applicable.

## Abbreviations

Aβ: Amyloid-β
AD: Alzheimer’s disease
BBB: Blood–brain barrier
Bregs: Regulatory B cells
CNS: Central nervous system
CSF: Cerebrospinal fluid
CXCL12: C-X-C motif chemokine ligand 12
CXCR4: Chemokine (C-X-C motif) receptor 4
C6-Luc: C6 glioma cells that luciferase reporter-gene labeled
DCs: Dendritic cells
DDS: Drug delivery systems
DOX: Doxorubicin
EVs: Extracellular vesicles
GDNF: Glial cell line-derived neurotrophic factor
ICAM-1: Intracellular adhesion molecule-1
ICAM-2: Intracellular adhesion molecule-2
IL-1β: Interleukin-1β
LFA-1: Lymphocyte function-associated antigen-1
MCAO: Middle cerebral artery occlusion
MS: Multiple sclerosis
NETs: Neutrophil extracellular traps
NK: Natural killer
PD: Parkinson’s disease
PSGL-1: P-selectin glycoprotein ligand-1
PTX: Paclitaxel
TAMs: Tumor-associated macrophages
TANs: Tumor-associated neutrophils
TJs: Tight junctions
TNF-α: Tumor necrosis factor-α
TME: Tumor microenvironment
TPP: Triphenylphosphine cation
Tregs: Regulatory T cells
VCAM-1: Vascular cell adhesion molecule-1
VLA-4: Very late antigen-4
